# Myo-Inositol Levels in the Dorsal Hippocampus Serve as Glial Prognostic Marker of Mild Cognitive Impairment in Mice

**DOI:** 10.3389/fnagi.2021.731603

**Published:** 2021-11-12

**Authors:** Tim Ebert, Daniel E. Heinz, Suellen Almeida-Corrêa, Renata Cruz, Frederik Dethloff, Tibor Stark, Thomas Bajaj, Oriana M. Maurel, Fabiola M. Ribeiro, Silvio Calcagnini, Kathrin Hafner, Nils C. Gassen, Christoph W. Turck, Benoit Boulat, Michael Czisch, Carsten T. Wotjak

**Affiliations:** ^1^Research Group Neuronal Plasticity, Max Planck Institute of Psychiatry, Munich, Germany; ^2^Research Group Neurohomeostasis, Department of Psychiatry and Psychotherapy, University Hospital Bonn, Bonn, Germany; ^3^Max Planck School of Cognition, Leipzig, Germany; ^4^Proteomics and Biomarkers, Max Planck Institute of Psychiatry, Munich, Germany; ^5^Department of Pharmacology, Faculty of Medicine, Masaryk University, Brno, Czechia; ^6^Scientific Core Unit “Neuroimaging”, Max Planck Institute of Psychiatry, Munich, Germany; ^7^Department of Translational Research in Psychiatry, Max Planck Institute of Psychiatry, Munich, Germany; ^8^Central Nervous System Diseases Research (CNSDR), Boehringer Ingelheim Pharma GmbH & Co. KG, Biberach an der Riss, Germany

**Keywords:** spatial memory, biomarker, MRS, MCI, glia, astrocytes, microglia, myo-inositol

## Abstract

Dementia is a devastating age-related disorder. Its therapy would largely benefit from the identification of susceptible subjects at early, prodromal stages of the disease. To search for such prognostic markers of cognitive impairment, we studied spatial navigation in male BALBc vs. B6N mice in combination with *in vivo* magnetic resonance spectroscopy (^1^H-MRS). BALBc mice consistently showed higher escape latencies than B6N mice, both in the Water Cross Maze (WCM) and the Morris water maze (MWM). These performance deficits coincided with higher levels of myo-inositol (mIns) in the dorsal hippocampus before and after training. Subsequent biochemical analyses of hippocampal specimens by capillary immunodetection and liquid chromatography mass spectrometry-based (LC/MS) metabolomics revealed a higher abundance of glial markers (IBA-1, S100B, and GFAP) as well as distinct alterations in metabolites including a decrease in vitamins (pantothenic acid and nicotinamide), neurotransmitters (acetylcholine), their metabolites (glutamine), and acetyl-L-carnitine. Supplementation of low abundant acetyl-L-carnitine via the drinking water, however, failed to revert the behavioral deficits shown by BALBc mice. Based on our data we suggest (i) BALBc mice as an animal model and (ii) hippocampal mIns levels as a prognostic marker of mild cognitive impairment (MCI), due to (iii) local changes in microglia and astrocyte activity, which may (iv) result in decreased concentrations of promnesic molecules.

## Introduction

Today approximately 50 million people worldwide suffer from dementia, which renders this neurodegenerative disease one of the major causes of cognitive impairment, immobility and dependency among elderly people. The incidence of dementia is expected to even further increase over the next decades, with an estimated 152 million people affected by this disorder worldwide in 2050 (Patterson, 2018). Despite intense research (Campisi et al., 2019; Cummings et al., 2019), none of the tested therapeutic agents has been shown to prevent disease progression or reverting it (Annear et al., 2015). It is conceivable that the interventions simply came too late when neuronal degeneration had surpassed a certain threshold (Aisen et al., 2017; Weller and Budson, 2018). Therefore, patients have to be identified at early, ideally at prodromal stages of the disease, in order to permit preventive interventions (Hampel et al., 2014). Preferably, such attempts should include longitudinal studies on prognostic markers, which directly relate to the pathology of dementia (e.g., cognitive changes and altered neurochemistry/anatomy of distinct brain structures) rather than surrogate markers (e.g., lifestyle factors, blood analytes, etc.). One interesting concept in this context is the diagnosis of mild cognitive impairment (MCI). MCI is defined as an intermediate stage of cognitive impairment that may evolve into dementia (Petersen, 2004). However, MCI patients are still able to handle their daily routine and preserve general cognitive and functional abilities.

Cognition is a complex process with many fold aspects, ranging from sensory perception to attention, motivation, memory formation, executive functions (e.g., planning, flexibility, inhibitory control, and decision making) and motor output (Keeler and Robbins, 2011). It is assumed that cognitive functioning is maintained by various compensatory mechanisms including increased mental effort during aging until exhaustion of cognitive reserve capacities. Only then cognitive deficits emerge (Whalley et al., 2004; Tucker and Stern, 2011; Grady, 2012). Therefore, MCI is a heterogeneous syndrome with variable progression of the disease, which makes it difficult to identify reliable predictive and valid biomarkers. One promising approach employs ^1^H-magnetic resonance spectroscopy (^1^H-MRS), as a potent translational tool, measuring non-invasively metabolite concentrations in brain structures implicated in cognition such as prefrontal cortical areas or hippocampus formation (Sigurdsson and Duvarci, 2015). In this context, a brief survey of the literature revealed two consistent observations in MCI patients: (1) a reduction of combined *N*-acetylaspartate (NAA) and *N*-acetyl-aspartyl-glutamate (NAAG) levels and (2) an increase of myo-inositol (mIns) levels (Franczak et al., 2007; Wang et al., 2009; Chen et al., 2016). However, human studies have several limitations, including difficulties to perform longitudinal measurements within the same subjects and the lack of insight into biological processes, which may underlie the changes in metabolite profile. Even though NAA + NAAG levels are believed to be markers of neuronal integrity (Castellano et al., 2012) and mIns a marker of glial cells (Brand et al., 1993) there is only sparse evidence in support of these markers from combined *in vivo* ^1^H-MRS measurements and *ex vivo* analyses of neuronal *vs.* glial proteins.

The present study set out to substantiate the link between cognitive performance, hippocampal levels of NAA + NAAG and mIns and markers of neuronal *vs.* glial cells. To this end we compared spatial learning capabilities of two inbred mouse lines (C57BL/6N vs. BALB/c) with known differences in cognitive performance (Zaharia et al., 1996; Francis et al., 2003) in the simpler Water Cross Maze (WCM; Kleinknecht et al., 2012) and the more challenging Morris water maze (MWM; Morris, 1984) tasks. We combined repeated behavioral testing with *in vivo* ^1^H-MRS measurements in the dorsal and ventral hippocampus, *ex vivo* measurements of neuronal postsynaptic density protein 95 (PSD95) as a postsynaptic marker of excitatory synapses (Hering and Sheng, 2001), activated microglia (IBA-1; Ito et al., 1998) and astrocytic markers (S100B; Schroeter and Steiner, 2009; glial fibrillary acidic protein; Zhang et al., 2019) and mass spectrometry-based analyses of metabolite levels. Finally, we studied behavioral consequences of supplementation with one of the low abundant metabolites, acetyl-L-carnitine (LAC), via the drinking water. Overall, our data suggest BALBc mice as a model for MCI, and mIns levels in the dorsal hippocampus as prognostic marker of MCI-like changes. Moreover, mIns levels seem to reflect glial cell activity, which might be involved in the pathogenesis of dementia. Supplementation of low abundant metabolites, however, did not revert the cognitive deficits.

## Materials and Methods

### Animals

Male C57BL/6NCrl (B6N) and BALB/cAnNCrl (BALBc) were obtained from Charles River (Bad Sulzfeld, Germany) at an age of 6 weeks and housed in groups of 3–4 in IVC cages (Greenline, Techniplast) under SPF conditions (23°C ± 4°C and 50% humidity ± 10%) with food and water *ad libitum* and a 12 h:12 h light-dark cycle (lights on: 08:00 h). Experiments were performed during the light phase of the light-dark cycle (i.e., between 09:00 and 17:00 h). The cages were equipped with bedding and rodent tunnel (4.5 cm × 4 cm, diameter: 30 mm; ABEDD, Vienna, Austria). Animals had at least 10 days to recover from transportation and to adapt to the new holding conditions before the experiments started. Animals of a given experiment were delivered and tested at the same time in order to keep environmental factors constant. In case of ^1^H-MRS imaging and WCM training, mice were isolated at least 1 week before starting the experiments and individually housed thereafter. Otherwise, mice were kept in groups of 3–4. All experimental procedures have been approved by the Government of Upper Bavaria (ROB-55.2-2532.Vet_02-17-224). All efforts were taken to reduce animal numbers and to minimize animal suffering.

### Behavioral Procedures

#### Water Cross Maze

Mice were trained in the WCM using a hippocampus-dependent place learning protocol (Kleinknecht et al., 2012; Reichel et al., 2014, 2017) essentially as previously described (Kleinknecht et al., 2012). In brief, the plus-shaped maze was made out of clear, transparent plexiglas with four identical arms (length and width of 50 cm × 10 cm). Arms were oriented according to the cardinal directions and were designated as N-, E-, W-, and S-arm. Every day, the maze was filled with fresh tap water (22°C ± 1°C) up to a height of 11 cm, and a transparent squared platform of 10 cm height and 8 cm × 8 cm surface area was placed at the end of either the E- or the W-arm (equally distributed between the two strains but kept constant for each individual). Mice were kept in the holding room adjacent to the training room by a door (for further description of the experimental room see MWM). For each trial, mice were individually carried to the training room and allowed 2–3 min to adapt to the low light conditions (<15 lux) before insertion into the pool facing the end wall of the start arm with the experimenter staying behind the start arm. Mice were removed from the pool once they had climbed onto the platform. If they had failed to do so within 30 s, they were guided to the platform before removal from the maze, and a latency of 31 s was noted. After each trial, mice were returned to the adjacent holding room and placed at safe distance of a heating lamp on the bench until starting the next trial.

For each training session, animals underwent six trials per day over a period of five consecutive days, with an inter-trial interval of approximately 10 min per day. The platform position was kept constant at a fixed position for each individual animal. The starting position was either the N- or the S-arm aligned in a pseudorandomized order with a total of 3 starts from N and 3 from S per day. For each trial, the WCM was turned into a T-maze by blocking the arm opposing the start arm. If an animal showed floating behavior, the experimenter “woke it up” by creating a loud noise (e.g., by snapping the gloves) and counted the trial as “floating trial.”

The following parameters were used to evaluate spatial learning and memory: (i) latency to reach the platform – averaged over the 6 trials per day, (ii) latency score as the sum of escape latencies over all trials, (iii) accuracy representing the number of correct trials per training day during which mice directly swam to the platform without returning to the start arm or entering the wrong arm (expressed as percentage of the 6 trials per day), (iv) accuracy score representing the average of accuracy over the course of the 5 training days, (v) number of accurate learners representing the number of mice which performed ≥5 (out of 6) correct trials per day, expressed as a percentage of the total number of mice per group, (vi) number of floating trials per day, and (vii) floating score representing the total number of floating trials over the five training days.

#### Morris Water Maze

The test was performed in a circular white pool with a diameter of 150 cm and 41 cm high walls that was placed on a table so that the edge of the wall was elevated 110 cm above the floor. The pool was filled with fresh tap water to a height of 33 cm on the first experimental day. The water temperature was maintained at 22 ± 1°C throughout the trials by addition of warm water. The pool was localized in the middle of a cubic-like room (W309 cm × L357 cm × H283 cm) which contained prominent customized landmarks at the walls (rectangular, triangular and circular posters with different black/white patterns, fixed 170 cm above the floor), but no windows or additional prominent cues. The computer for video tracking was placed in the SW corner and a sink was located in the NE corner, both not visible to the animals from the pool. The room was illuminated by indirect light with the help of two spot lights facing the wall and highlighting the customized landmarks, resulting in 11.5 lux at the water surface level. The circular 8 cm × 8 cm escape platform of clear acryl plexiglas was placed in a fixed position in the NW quadrant 1 cm beneath the water surface and 35 cm away from the wall.

Mice were transported cage-wise from the holding to the adjacent training room, and the cage was left in place until the 4th trial was completed. Thereafter, they were moved back to the holding room and placed at a safe distance of a heating lamp for 10 min. Each animal underwent 4 trials per day with varying starting positions in pseudorandomized order over a period of 7 days. The daily inter-trial interval was 10 min. The starting positions were assigned in a random order out of 6 positions evenly distributed along the perimeter of the pool. For each trial, mice were gently placed on the water surface facing the wall, and the experimenter took a seat in fixed position. If an animal climbed onto the platform, it remained there for additional 5 s until the experimenter brought the animal back to its home cage with the help of a metal grid fixed to a stick. If an animal did not find the platform within 60 s, the experimenter guided the animal to the platform with the stick without touching the mouse and noted 61 s as escape latency.

Each trial was recorded and analyzed by ANY-MAZE (v5.26; Stoelting, Dublin, Ireland). We assessed the following main performance parameters: (i) escape latencies averaged over the 4 trials per day, (ii) latency score as the sum of all escape latencies over the 7 days training period, (iii) the number of trials during which mice did not reach the platform within 60 s (failures), and (iv) swimming path length averaged over the 4 trials per day.

On Day 8, animals performed a 60-s probe trial (PT) during which the platform was removed from the maze, and the animals were started from the quadrant opposite to the target quadrant. The video tracking software divided the pool into 4 quadrants, and we assessed (v) the swimming path length per quadrant as a percentage of the total swimming path length.

### *In vivo* ^1^H-Magnetic Resonance Spectroscopy

Mice were anesthetized using Isoflurane (Isofluran CP^®^, CP-Pharma^®^, Burgdorf, Germany). Anesthesia was introduced at a concentration of 2.0% (air flow 1.3 l/min) and maintained at 1.5–2.0% throughout the scanning procedure. Animals were placed in prone position on an animal bed with a heating pad (water bath, Haake S 5P, Thermo Fisher Scientific, Waltham, United States) and a pressure pillow to monitor respiration (adjusted to 80–120 bpm by the isoflurane concentration). The head was stereotactically fixed to reduce motion artifacts. Ophthalmic ointment (Bepanthen^®^ Bayer AG, Leverkusen, Germany) was applied on their eyes to prevent damage due to drying during scanning. Body temperature was recorded and monitored using a rectal probe and kept at the range of 36.5–37.5°C. After the successful acquisition, anesthesia was discontinued and the mouse was returned to its home cage. Recovery of the animals was monitored. Total time of anesthesia reached 65–90 min per mouse.

Animals were scanned with a 9.4T BRUKER Biospec 94/20 system using Paravision software (Paravision 6.0.1, Bruker, Ettlingen, Germany). For most ^1^H-MRS experiments (MRS 1, MRS 2a and MRS 3) data were collected using a two channel transmit/receive cryocoil. Due to technical reasons, for the MRS experiment MRS 2b, data were collected using a whole body coil for transmission and a room temperature 2 × 2 array surface coil for signal detection.

#### Magnetic Resonance Spectroscopy Acquisition

Twenty-three animals were included in Experiment 1 (12 BALBc, 11 B6N). Due to technical issues, MRS data from one BALBc and one B6N mouse were not successfully acquired.

In Experiment 2a, 35 animals were scanned before completion of the third training session [23 BALBc, 12 B6N; for two of the BALBc animals, no spectroscopy data could be collected due to failure to reach appropriate shim values and two mice died before the third round of training (T3)].

For Experiment 2b, animals were scanned after completion of the third training session. Five animals had to be excluded due to mortality or technical reasons. Spectra were recorded in 18 BALBc and 12 B6N.

In Experiment 4, after the swimming BALBc mice treated with LAC (*n* = 12) or tap water (*n* = 12) were scanned. One LAC and two control mice were excluded due to inappropriate shim values.

For MRS localization, a 2D T2-weighted structural image was acquired with 25–30 slices (slice thickness 0.25 mm; 0.1 mm gap; image matrix 384 × 384 voxel; TR = 328 or 348 ms; TE = 3.2 ms; flip angle 30°; FOV = 20 mm × 20 mm). PRESS spectroscopy (TR = 5,000 ms; TE = 16.5 ms; 128 averages; voxel size 2.5 mm × 1.5 mm × 1.5 mm) was run for the right and left dorsal (Experiments 1, 2 and 4) as well as ventral HPC (Experiment 2).

The shimming of first and second order shims was performed with MAPSHIM after recording a field map. Before acquisition of the final spectrum, shimming was initially using a one shot unsuppressed water STEAM spectrum (TR = 2,500 ms; TE = 3 ms; TM = 10 ms) with the same spatial and spectral parameters as the later PRESS spectrum. We assessed the line width of the water peak at height 10% and 50% (50% – Full width at half maximum, FWHM) from this spectrum, for each and every individual, and voxel location. We aimed at achieving a FWHM below 20 Hz and the 10% width below 60 Hz before starting the final acquisition.

To compensate for frequency drifts during the collection of the individual free-induction decays (FIDs), drift compensation based on navigator echoes (as implemented in ParaVision 6.0.1) was included. In addition, we used BRUKER’s eddy current correction during spectrum reconstruction. Eddy current correction, as well as the referencing of the metabolite concentrations to the voxel’s water content, relied on an undistorted water spectrum which was acquired with identical MRS parameters as the final spectrum, using the PRESS sequence (the only difference between these two acquisitions was that no water suppression was applied, the receiver gain was adjusted, and only a single FID was recorded).

For the final spectrum, we used VAPOR for water suppression. The total time for each location of the MRS experiment was 10 min 40 s.

#### Magnetic Resonance Spectroscopy Analysis

Spectral analysis and estimation of metabolic concentrations was performed with LCModel 6.3^1^. The 9.4T basis spectra included in LCModel was used (also including the default simulated basis spectra of macromolecules and lipids), with an analysis window ranging from 4.3–0.2 ppm. In some of our hippocampal spectra, larger lipid contamination was observed. For these data, the ‘tumor’ option was used, which allows for a proper automated phasing of the spectrum in the presence of strong lipid signals. As the total creatine (tCr; sum of creatine and phosphocreatine) concentrations, scaled to the voxel’s internal water concentration, did not differ between groups, we chose to present all metabolite concentrations referenced to the total creatine signal as an internal standard. Under *in vivo* conditions, some resonances cannot be reliably distinguished from each other. This applies to NAA and NAAG, which are therefore reported only as combined NAA + NAAG, as well as for Cr and PCr (reported as tCr).

#### Magnetic Resonance Spectroscopy Quality Assurance

To be included in the final statistical analysis, spectra had to meet the following quality criteria: (i) successful fit of the spectrum, i.e., no distinct spectral lines obviously not properly fitted by the model (see Supplementary Figure 2); (ii) line width estimate (FWHM) as derived by LCModel ≤ 0.118 ppm; (iii) Signal-to-Noise (SNR) ≥ 7 for the cryocoil and SNR ≥ 3 for the room temperature coil (SNR is defined as the ratio of the maximum in the spectrum-minus-Baseline in the analysis window to twice the rms residuals); (iv) Cramér-Rao lower bound (CRLB, expressed in% of the estimated concentrations) < 15% [for (ii)–(iv)], see Text Footnote 1). The descriptive statistics for the individual sub-experiments is shown in Supplementary Table 1. Representative spectra for both the dorsal and ventral hippocampal voxel can be found in Supplementary Figure 1, along with a graphical depiction of the voxel position. Please refer to the Supplementary Material for a table summarizing all MRS parameters in line with experts’ consensus recommendations (Lanz et al., 2020; Lin et al., 2021).

### Capillary Immunoblotting

For protein extraction, specimens from the dorsal hippocampus were mixed with RIPA buffer [150 mM NaCl, 1% IGEPAL CA-630, 0.5% sodium deoxycholate, 0.1% SDS, 50 mM Tris (pH 8.0)] supplemented with protease inhibitor (Merck Millipore, Darmstadt, Germany), benzonase (Merck Millipore), 5 mM DTT (Sigma-Aldrich, Munich, Germany), and phosphatase inhibitor (Roche, Penzberg, Germany) cocktail.

Proteins were separated and analyzed by capillary electrophoresis on Wes^TM^ (ProteinSimple; San Jose, CA, United States) using the 12–250 kDa cartridges. The following primary antibodies were utilized for immunodetection: anti-GFAP (1:100, Cell Signaling Technology, #80788), anti-S100B (1:100, Cell Signaling Technology, #9550), anti-IBA1 (1:100, FUJIFILM Wako Pure Chemical Corporation, 019-19741), anti-PSD95 (1:100, Cell Signaling Technology, #3450), anti-β-actin (1:300, Cell Signaling Technology, #4970). Anti-Rabbit detection module (ProteinSimple, San Jose, CA, United States) was used detecting primary antibodies. Expression levels were normalized to the intensity of β-actin.

### Metabolomic Analyses

#### Sample Preparation

Plasma samples were collected in EDTA tubes (Sarstedt, Nümbrecht, Germany) after decapitation under anesthesia. Plasma metabolite samples were extracted by adding 200 μl MeOH to 50 μl plasma. Samples were vortexed for 1 h at 25°C in a ThermoShaker and subsequently centrifuged for 10 min at 16,000^∗^*g*. 200 μl supernatant was transferred into an Eppendorf tube, dried in a vacuum centrifuge and stored at −20°C until measurement. Tissue sample metabolites were extracted by adding 300 μl cold 80% MeOH to 1 mg tissue. Tissue samples were ground with a pistil and incubated in a shaker for 1.5 h at 4°C. Samples were centrifuged for 10 min at 16,000^∗^*g*. 200 μl supernatant was transferred into an Eppendorf tube, dried in a vacuum centrifuge and stored at −20°C until measurement. For LC-MS measurements, samples were dissolved in water and diluted 1:4 with ACN to a final concentration of 0.05 μg/μl. For analysis 2 μl were injected into the injection loop of the HPLC.

#### Liquid Chromatography–Mass Spectrometry Metabolite Analysis

Liquid chromatography–mass spectrometry analysis was performed with the following equipment: Agilent Technologies 1100 HPLC system (Agilent Technologies; Santa Clara, CA, United States) was connected to a Bruker Impact II TOF MS system (Bruker Corporation; Billerica, MA, United States). Both systems were controlled by Bruker Hystar 3.2 software. Samples were fractionated with a two eluent gradient (eluent A: 20 mM ammonium formate, pH 3; eluent B: ACN with 0.1% formic acid), using a flow rate of 500 μl^∗^min^–1^ and a Waters XBridge Amide 3 mm × 100 mm column with 2.6 μm particle size and 200 Å pore size (Waters Corporation, Milford, MA, United States) and a Waters XBridge Amide guard column. Both columns were heated to 25°C in the LC oven. The following gradient was used. Isocratic elution with 95% eluent B for 2 min. The eluent B was decreased to 65% over a course of 23 min followed by a drop to 50% in 1 min and maintained at 50% for 3 min. Next eluent B was increased to 95%, and the column equilibrated for 26 min. For positive mode (ESI+), capillary voltage was set to 4,000 V. The dry gas N_2_ was heated to 220°C with a flow rate of 10 l^∗^min^–1^. The pressure of the nebulizer was maintained at 3.5 bar. The profile data was assessed by Bruker Compass 1.9 software with a spectra rate of 1 Hz (full scan) and a mass range from 20–1,300 m/z. Internal calibration of the mass accuracy was performed by injecting sodium formate clusters prior to each run and adjusting the results with Bruker DataAnalysis 4.4 software. Data files were converted to mzXML with MSConvert (ProteoWizard, Palo Alto, CA, United States). MZmine2 (Pluskal et al., 2010) was used for data pre-processing. Chromatographic peaks were created with the ADAP module (Chowdhury et al., 2009). Deisotoped peak lists were annotated according to retention time and exact mass using an inhouse database. For statistical analysis the final compound list was exported to a CSV file and imported to Microsoft Excel. Data was normalized to tissue fresh weight and Students *t*-test was calculated.

#### Pharmacological Treatment

Acetyl-L-carnitine (LAC; Sigma-Aldrich, Taufkirchen, Germany) was added to the drinking water up to a concentration of 0.3% as previously reported (Cherix et al., 2020). Each cage with four animals had *ad libitum* access to one drinking bottle. Non-treated controls had regular drinking water. The treatment lasted from 7 days prior to the first day of testing until the end of the experiment. Fluid intake was monitored on a regular basis.

### Experiments

#### Experiment 1

Eight weeks old, experimentally naïve B6N (*n* = 12) and BALBc mice (*n* = 12) were trained in the WCM for 5 days, followed by ^1^H-MRS measurements starting 1 week later (MRS 1). At the end of scanning, mice (3 months old) were killed by an overdose of isoflurane, and brains were collected and stored at −80°C. Bilateral specimens from the dorsal hippocampus were used for capillary immunoblotting (Wes, ProteinSimple).

#### Experiment 2

New groups of experimentally naïve B6N (*n* = 12) and BALBc (*n* = 24) mice underwent ^1^H-MRS measurements at an age of 9–10 weeks (MRS 2a), followed by three 5-days WCM training sessions at 4-month intervals corresponding to an age of 12–13 (T1), 29–30 (T2) and 47–48 (T3) weeks. After a second ^1^H-MRS measurement at the age of 49–50 weeks (MRS 2b), mice were sacrificed, and brains were collected and stored at −80°C for later analysis. Parts of the bilateral specimens from the dorsal hippocampus were used for ProteinSimple measurements, the other part for metabolite analyses. Due to technical problems and mortality, only 19 BALBc mice finished the last scan.

#### Experiment 3

Ten weeks old, experimentally naïve B6N (*n* = 9) and BALBc (*n* = 10) mice underwent a 7-day MWM training followed by a probe trial at day 8.

#### Experiment 4

New groups of 10 weeks old, experimentally naïve BALBc mice were either treated with LAC dissolved in water (*n* = 12) or with tap water (*n* = 12). After 7 days of treatment both groups of mice performed MWM training. Thereafter, they were scanned with ^1^H-MRS (MRS 3) and dorsal hippocampus specimens as well as plasma samples were analyzed for LAC content.

### Statistics

Data were analyzed by *t*-test, Mann–Whitney *U*-test, ANOVA for repeated measurements or Chi-square test, followed by *post hoc* test as indicated in the text. In case of 2-group comparisons, we additionally calculated the effect sizes (Cohen’s *d*). If not stated otherwise, data are shown as mean ± SEM. Statistical significance was accepted if *p* < 0.05.

*In vivo* imaging and metabolomics data were analyzed as described in the respective Section “Materials and Methods”.

## Results

### Higher Myo-Inositol Levels in BALBc Were Associated With Increased Escape Latencies but Unimpaired Accuracy in the Water Cross Maze (Experiment 1)

**FIGURE 1:**
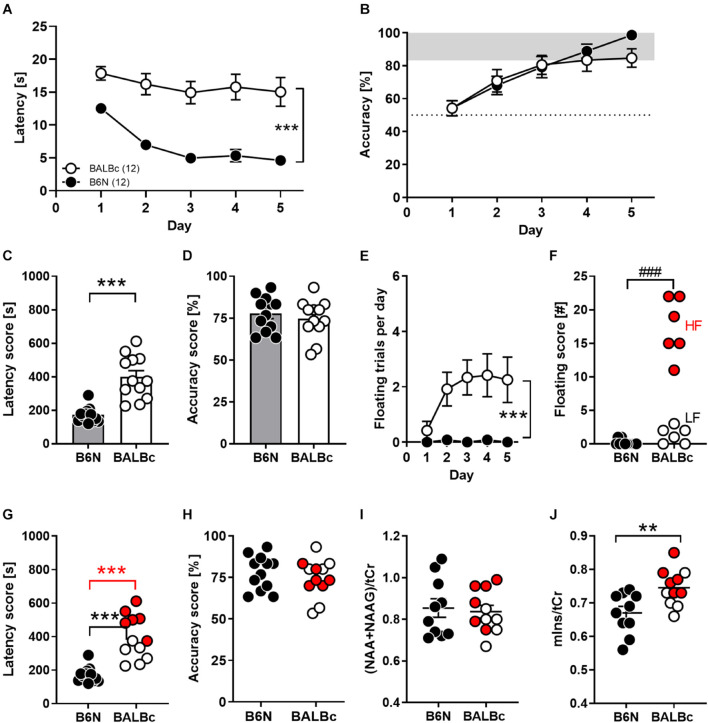
Performance deficits in spatial learning in the Water Cross Maze (WCM) coincided with increased mIns levels in the dorsal hippocampus of BALBc mice. Eight weeks old B6N (*n* = 12) and BALBc (*n* = 12) mice were trained in the WCM to locate a hidden platform which was kept at a fixed position. Thereafter, metabolite levels were measured with *In vivo* ^1^H-MRS in the dorsal hippocampus. **(A,C)** BALBc mice showed significantly greater escape latencies, but **(B,D)** no differences in accuracy throughout training. **(E,F)** BALBc showed a higher incidence of floating behavior, regardless if high levels of floating (HF) or low levels of floating (LF). **(G)** HF and LF BALBc mice showed higher escape latencies compared to B6N, **(H)** without affecting accuracy. **(I,J)** *In vivo* ^1^H-MRS measurements in the dorsal hippocampus after training revealed significantly higher levels of myo-inositol (mIns), but not NAA + NAAG compared to B6N. Interestingly, this effect was particularly pronounced in HF BALBc mice [tCr = total creatine (creatine + phosphocreatine)]. **p < 0.01, ***p < 0.0001 [main effect of strain in 2-way ANOVA, 1-way ANOVA followed by Tukey’s *post hoc* test (g) or *t*-test]; ^###^*p* < 0.001 (Mann–Whitney *U*-test).

To compare cognitive abilities of both mice strains, 8 weeks old B6N and BALBc mice were trained in the WCM using the place-learning protocol over the course of 5 days. This task enables to monitor hippocampus-dependent parameter (i.e., accuracy) in additions to escape latencies over the course training (Kleinknecht et al., 2012). Moreover, due to its simplicity, mice more easily reach the platform compared to training in the Water maze, which reduces the stress load and frustration. Nevertheless, it took BALBc mice significantly longer to navigate to the platform than B6N mice, which showed a decrease in escape latencies from day to day (factor *Strain*: *F*_1,__110_ = 115.9, *p* < 0.0001; 2-way ANOVA for repeated measures; Figure 1A). There was virtually no overlap between the two groups if the sum of all escape latencies (30 trials) was considered (*t*_22_ = 5.757, *p* < 0.0001; Cohen’s *d*: 2.2; Figure 1C). In stark contrast, as shown by the accuracy levels, both strains learned similarly well to directly navigate to the platform position (factor *Strain*: *F*_1,__110_ = 0.792, *p* = 0.376; *Strain* × *Day* interaction: *F*_4,__110_ = 0.793, *p* = 0.532; Figure 1B). This also became evident if the averaged accuracy scores were evaluated (*t*_22_ = 0.690, *p* = 0.497; Figure 1D). By day 5 of training, 12/12 B6N (100%) and 9/12 BALBc mice (75%) had reached the learning criterion of at least 5 daily correct trials out of 6 (data not shown).

The discrepancy between escape latencies and accuracy can be explained in part by differences in coping styles due to the stressful learning environment. Whereas approximately 50% of BALBc mice showed a high prevalence of floating behavior from day to day, floating was virtually absent in B6N mice (Mann–Whitney-*U*-test: *p* = 0.0002; Figures 1E,F). Nevertheless, not only high-floating (605 ± 39 s; *t*_16_ = 11.48, *p* < 0.0001) but also low-floating BALBc mice (352 ± 29 s; *t*_16_ = 4.815, *p* = 0.0002) had higher total escape latency scores than B6N mice (207 ± 16 s; Figure 1G), whereas accuracy levels were unrelated to the incidence of floating (statistics not shown; Figure 1H). Consequently, BALBc mice revealed general deficits in performance that cannot be explained solely by a higher prevalence for increased floating behavior.

Starting 1 week after training, mice underwent ^1^H-MRS for the assessment of NAA + NAAG and mIns levels within the dorsal hippocampus. Whereas levels of NAA + NAAG were indistinguishable between the two strains (*t*_19_ = 0.331, *p* = 0.744; Cohen’s *d*: 0.1; Figure 1I), BALBc mice showed significantly higher mIns levels than B6N mice (*t*_19_ = 2.997, *p* = 0.0074; Cohen’s *d*: 1.2; Figure 1J). Remarkably, BALBc mice with higher, but not those with lower prevalence of floating behavior, showed significantly higher mIns levels than B6N mice (*F*_2,__18_ = 6.482, *p* = 0.0076; Tukey’s *post hoc*: B6N vs. BALBc_HF *p* = 0.0056; Figure 1J), suggesting that mIns levels reflect differences in coping styles.

### BALBc Mice Showed Persistent Deficits in Spatial Learning, but Not Memory, in the Water Cross Maze Over Time That Coincided With Persistently Higher Myo-Inositol Levels (Experiment 2)

In a second step, we aimed to investigate the trajectory of cognitive performance of both mice strains with increasing age.

**FIGURE 2:**
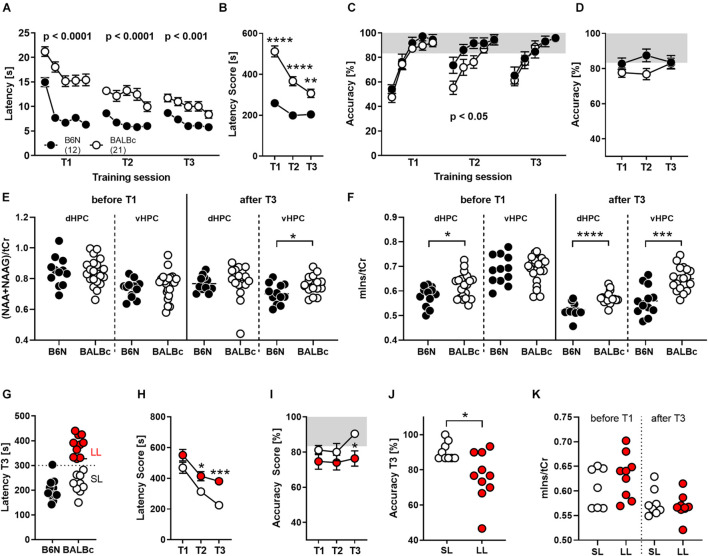
BALBc mice showed persistent deficits in WCM learning, but not memory, over time coinciding with increased mIns levels both in the beginning and the end of the experiment. To investigate the performance of both strains with increasing age, B6N (*n* = 12) and BALBc (*n* = 19) mice were repeatedly trained in the WCM to find a hidden platform in a fixed position at an age of 3 months (T1), 7 months (T2), and 12 months (T3). Before T1 and after T3, ^1^H-MRS measurements have been performed in both mice strains. **(A)** BALBc mice showed increased escape latencies throughout training, whereby **(B)** the performance of the two strains started to converge toward the end of training. **(C,D)** BALBc mice also showed a deficit in accuracy compared to B6N (T2). This strain difference, however, was transient. **(E)** As assessed by repeated ^1^H-MRS (before the start and after completion of the whole experiment), there were no strain differences in NAA + NAAG at the level of the dorsal and ventral hippocampus except for the late time point. **(F)** In contrast, mIns levels differed before and after repeated training in the dorsal hippocampus (dHPC) or after training in the ventral hippocampus (vHPC). **(G)** We assigned the BALBc mice to two groups, based on their escape latency shown during T3, using an arbitrary threshold of 300 s to distinguish between long-latency (LL) and short-latency (SL) mice. **(H)** LL and SL mice differed in escape latencies already during T2 and **(I,J)** in accuracy during T3. **(K)** These effects, however, were not mirrored by differences in mIns levels [tCr = total creatine (creatine + phosphocreatine)]. *p < 0.05, **p < 0.01, ***p < 0.001, ****p < 0.0001 (2-way ANOVA followed by Tukey’s *post hoc* test or *t*-test).

Therefore, we repeatedly trained new groups of BALBc and B6N mice at an age of 12–13 weeks (training 1, T1), 29–30 weeks (T2) and 47–48 weeks (T3) in the WCM, using the same place learning protocol as in Experiment 1. Again, BALBc showed significantly higher escape latencies not only during the first (T1; *Strain*: *F*_1,__31_ = 52.45, *p* < 0.0001; Figure 2A), but also the second (T2; *Strain*: *F*_1,__29_ = 32.95, *p* < 0.0001; Figure 2A) and the third (T3; *Strain*: *F*_1,__29_ = 13.83, *p* = 0.0009; Figure 2A) training episodes. Despite these consistent strain differences, escape latencies of the two lines tended to converge toward the end of repeated training (*Strain* × *Training episode*: *F*_2,__58_ = 12.21, *p* < 0.0001; Figure 2B). The decrease in escape latencies observed in BALBc mice (Figure 2B) contradicts the hypothesis that BALBc mice’s deficits simply result from deficits in stress coping or training motivation. In this case, we would have expected unchanged if not increased escape latencies over the course of training.

In agreement with our observations from Experiment 1, the deficits in spatial learning were not reflected by similar deficits in spatial memory. Despite the longer escape latencies, BALBc mice showed the same accuracy scores as B6N during T1 (*Strain*: *F*_1,__31_ = 1.221, *p* = 0.277; Figure 2C) and T3 (*Strain*: *F*_1,__29_ = 0.018, *p* = 0.895; Figure 2C). Only during T2 they showed significantly lower accuracy levels (*Strain*: *F*_1,__31_ = 4.723, *p* = 0.038; Figure 2C). This strain difference appears to be minor, since analysis of the accuracy levels over the course of repeated training failed to reveal significant strain differences (*Strain*: *F*_1,__29_ = 2.001, *p* = 0.167; *Strain* × *Training*: *F*_2,__58_ = 2.521, *p* = 0.0892; Figure 2D). The lack of strain differences at an age of 12 months (T3) seems to relate, at least in part, to an age-related decrease in spatial memory also in B6N mice, which becomes evident if accuracy levels during the first day of each training session are considered.

The repeated training episodes were embedded in two ^1^H-MRS measurements. This time we assessed NAA + NAAG and mIns levels in the dorsal and ventral hippocampus before the first training episode in young, experimentally naïve mice and after the last training when the animals are aged and experienced. In young, experimentally naïve mice we observed higher mIns levels in the dorsal hippocampus (*t*_31_ = 2.451, *p* = 0.020; Cohen’s *d*: 0.9; Figure 2F), whereas all other measures were similar between B6N and BALBc mice (statistics not shown; Figures 2E,F). In aged mice, we observed higher NAA + NAAG levels in the ventral hippocampus (*t*_27_ = 2.589, *p* = 0.015; Cohen’s *d*: 0.9; Figure 2E) and higher mIns levels in the dorsal (*t*_26_ = 4.768, *p* < 0.0001; Cohen’s *d*: 1.8; Figure 2F) and ventral hippocampus of BALBc mice (*t*_28_ = 4.499, *p* = 0.0001; Cohen’s *d*: 1.6; Figure 2F).

Once more, we could identify two subgroups of BALBc mice on the basis of the escape latencies shown during T3: “short latency” (SL) and “long latency” (LL) mice (Figure 2G). SL and LL mice already differed significantly during T2 (*Training*: *F*_1,__17_ = 15.19, *p* = 0.0012; Figure 2H), indicating a general trait for performance deficits in this strain. Remarkably, differences in escape latencies were reflected by differences in accuracy during the last training episode (*Training*: *F*_1,__17_ = 3.746, *p* = 0.07; T3: *t*_17_ = 2.289, *p* = 0.010, Cohen’s *d*: 1.2), but not earlier (Figures 2I,J). This implies that deficits in performance (as reflected by increased escape latencies) indeed coincide with impaired spatial memories in close to 1-year old BALBc mice.

The interindividual differences in escape latencies observed among BALBc mice were unrelated to mIns levels in the dorsal hippocampus before (*t*_14_ = 1.139, *p* = 0.274) and after repeated training (*t*_15_ = 0.552, *p* = 0.589; Figure 2K). Therefore, the previously observed putative relationship between mIns and floating behavior (Figure 1J) is not robust, in contrast to the strain difference in mIns levels between BALBc and B6N mice. This suggests that mIns levels are able to predict performance deficits in spatial learning at the strain but not individual level, at least in case of inbred mice.

### Increased Myo-Inositol Levels in BALBc Mice Coincided With an Increase in Glial Marker Proteins

**FIGURE 3:**
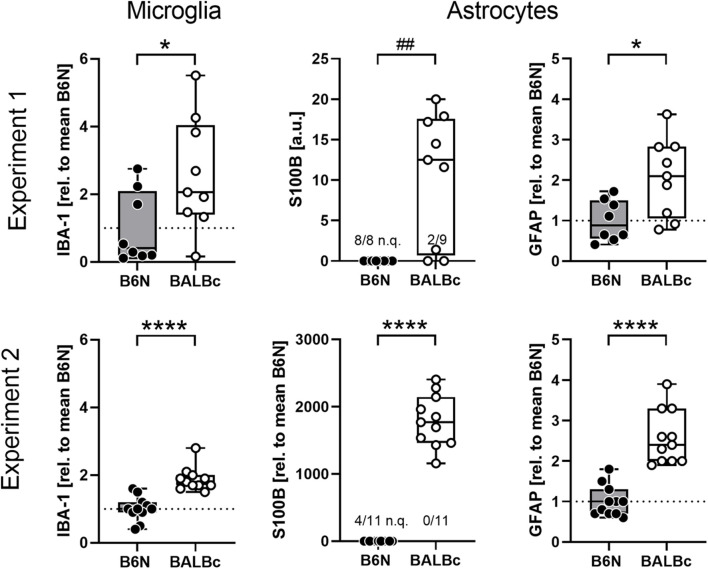
BALBc mice showed increased levels of glial markers in the dorsal hippocampus. Brain specimens were collected from the dorsal hippocampus of B6N and BALBc mice at the end of Experiments 1 and 2 corresponding to an age of 3 vs. 12 months. Protein simple measurements revealed elevated levels of microglia (IBA-1) and astrocytic (S100B, GFAP) markers in BALBc compared to B6N. If possible, data were normalized to the mean of B6N. In some cases, the signal was too weak for quantification (n.q.) which precluded normalization. *p < 0.05, ****p < 0.0001 (*t*-test), ^##^*p* < 0.01 (Mann–Whitney *U*-test).

In the search for cellular correlates of the line differences in mIns, we analyzed brain specimens from the dorsal hippocampus of the animals from Experiment 1 for glial marker proteins expression. We observed elevated levels of IBA-1 (*t*_15_ = 2.294, *p* = 0.0367, Cohen’s *d*: 1.0; Figure 3), S100B (*U*_44,__109_ = 8, *p* = 0.0023; was not quantifiable in 8/8 B6N, but only in 2/9 BALBc; chi^2^ = 10.58, *p* = 0.0011) and GFAP (*t*_15_ = 2.784, *p* = 0.0139, Cohen’s *d*: 1.3) in BALBc compared to B6N mice (Figure 3). Analysis of brain specimens from Experiment 2 revealed essentially the same strain differences with higher levels of IBA-1 (*t*_20_ = 5.760, *p* < 0.0001, Cohen’s *d*: 2.2), S100B (*t*_20_ = 15.39, *p* < 0.0001, Cohen’s *d*: 6.2; was not quantifiable in 4/11 B6N; chi^2^ = 4.889, *p* = 0.0270) and GFAP (*t*_20_ = 6.803, *p* < 0.0001, Cohen’s *d*: 2.8) in BALBc mice (Figure 3). This time we additionally analyzed PSD95, a postsynaptic marker of excitatory neurons, but failed to observe strain differences (*t*_20_ = 1.029, *p* = 0.316, Cohen’s *d*: 0.4; data not shown), suggesting a specific difference in glial proteins.

### BALBc Mice Showed Increased Escape Latencies but Intact Spatial Memory in the Morris Water Maze

**FIGURE 4:**
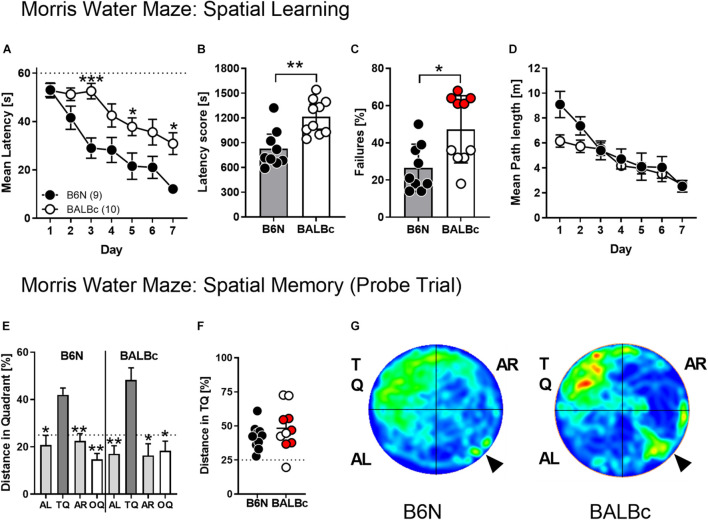
BALBc mice showed increased escape latencies but intact spatial memory in the MWM. To analyze the cognitive performance in a more demanding spatial navigation task, 10 weeks old B6N (*n* = 9) and BALBc (*n* = 10) mice were trained in the MWM over the course of 7 days with 4 training trials per day, followed by a probe trial at day 8 whereby the hidden platform was removed from the NW quadrant (TQ). **(A,B)** BALBc mice show longer escape latencies throughout training, which **(C)** is reflected by a higher number of trials in which the animals failed to reach the platform within 60 s (marked in red: subset of BALBc mice with particularly pronounced failure rate). The strain differences were not reflected if **(D)** the swimming path lengths during training and **(E–G)** the selectivity of spatial memory assessed in the probe trial were considered (AL, adjacent left quadrant; AR, adjacent right quadrant; OQ, opposite quadrant; TQ, target quadrant). *p < 0.05, **p < 0.01, ***p < 0.001 vs. other strain or TQ (1-way or 2-way ANOVAs followed by Tukey’s *post hoc* test or unpaired *t*-test).

The absence of deficits in spatial memory in the WCM that was observed despite the different performance (escape latency) and glial protein and metabolic marker levels, might be ascribed to the obvious simplicity of this learning task, which is sensitive only to major changes in hippocampus integrity (Kleinknecht et al., 2012; Reichel et al., 2014, 2017). Therefore, we trained new cohorts of 10 weeks old B6N and BALBc mice in the MWM, which requires a more complex cascade of switches between hippocampus-independent and -dependent behavioral strategies over the course of training (Ruediger et al., 2012; Garthe and Kempermann, 2013). Also, in the MWM, BALBc showed significantly increased escape latencies over the course of training (*Strain*: *F*_1,__17_ = 14.66, *p* = 0.0013; *Strain* × *Day*: *F*_6,__102_ = 2.302, *p* = 0.0396; Figure 4A) and if the total latency scores were considered (*t*_17_ = 3.828, *p* = 0.0013; Figure 4B; Cohen’s *d*: 1.7). In fact, BALBc mice failed to reach the platform within the 60-s limit in almost 50% of the trials (*t*_17_ = 2.846, *p* = 0.0112) with a bimodal distribution of the animals (Figure 4C).

Despite the strain differences in escape latencies, there were no significant differences in the swimming path length over the course of training (*Strain*: *F*_1,__17_ = 1.843, *p* = 0.1923; *Strain* × *Day*: *F*_6,__102_ = 1.939, *p* = 0.0817; Figure 4D). Importantly, BALBc mice developed a clear preference for the target quadrant in the probe trial (*F*_3,__27_ = 9.10, *p* = 0.0002; rmANOVA), similarly to B6N (*F*_3,__27_ = 10.15, *p* = 0.0001; Figure 4E) with no differences between the two strains (*t*_18_ = 1.069, *p* = 0.2991; Figures 4F,G) and the two subsets of BALBc mice which had differed in the failure rate during training (Figure 4F).

### BALBc Mice Showed Distinct Metabolite Differences in the Dorsal Hippocampus

**FIGURE 5:**
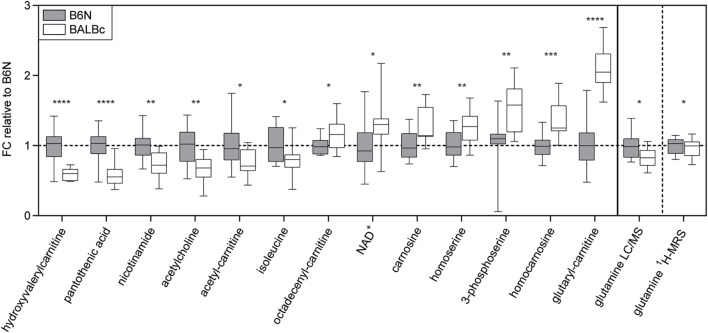
Metabolomics. Significant strain differences of metabolite levels in the dorsal hippocampus of B6N (*n* = 12) and BALBc mice (*n* = 15) from Experiment 2 revealed by mass spectrometry, aligned according to the effect sizes. Results are normalized to metabolite abundances in B6N mice, *y*-axis shows the fold change (FC). Glutamine ^1^H-MRS levels obtained before training in Experiment 2 were re-analyzed and are presented on the right panel of the figure. *p < 0.05, **p < 0.01, ***p < 0.001, ****p < 0.0001 (student’s *t*-test).

To complement the findings obtained by ProteinSimple (cf. Figure 3), we analyzed metabolite profiles in the dorsal hippocampus of mice from Experiment 2 using liquid chromatography–mass spectrometry (LC-MS). Among the metabolites analyzed, we observed a number of significant differences in BALBc compared to B6N mice (Figure 5). These differences were related to the lower abundance of vitamins (pantothenic acid, nicotinamide), acetyl-L-carnitine and neurotransmitters (acetylcholine) or their precursors (glutamine). The latter observation prompted us to re-analyze the ^1^H-MRS spectrograms from Experiment 2 in respect to glutamine levels: Indeed, we could observe significantly lower levels in BALBc compared to B6N also in these *in vivo* measures (Figure 5).

### Acetyl-L-Carnitine Treatment Did Not Improve Cognitive Performance of BALBc Mice

**FIGURE 6:**
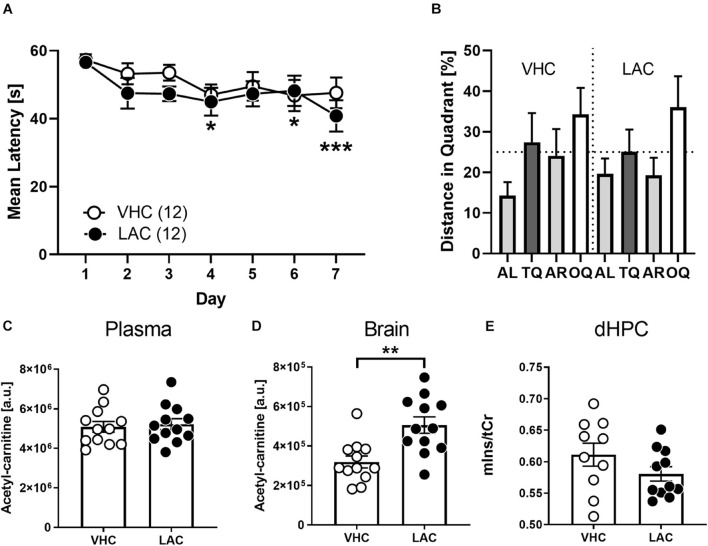
Supplementation of LAC did not invert cognitive deficits of BALBc mice. Ten weeks old LAC-treated BALBc (*n* = 12) and vehicle (VHC)-treated BALBc mice (*n* = 12) performed MWM training over the course of 7 days, followed by *In vivo*^1^H-MRS measurements. No consistent differences in spatial learning or memory were observed comparing **(A)** escape latencies or **(B)** distance in target quadrant. **(C)** While oral supplementation of LAC via the drinking water did not affect plasma LAC levels, **(D)** brain LAC levels were significantly elevated. **(E)** *In vivo*^1^H-MRS measurements revealed no differences in dorsal hippocampal mIns levels between both groups. [AL, adjacent left quadrant; AR, adjacent right quadrant; OQ, opposite quadrant; TQ, target quadrant; tCr = total creatine (creatine + phosphocreatine)]. *p < 0.05, **p < 0.01, ***p < 0.001 vs. other strain or TQ (2-way ANOVAs followed by Tukey’s *post hoc* test or unpaired *t*-test).

Our metabolomics analysis revealed significant differences in carnitine levels of BALBc compared to B6N mice. Particularly the reduced acetyl-carnitine content evoked our interest, since supplying acetyl-L-carnitine has shown beneficial effects in cognitively impaired humans (Montgomery et al., 2003) and animal models of chronic stress (Cherix et al., 2020). Therefore, we treated 10 weeks old BALBc mice with acetyl-L-carnitine via the drinking water and examined their behavioral performance in the MWM. Indeed, supplementing acetyl-L-carnitine led to increased acetylcarnitine levels in the HPC (but not blood plasma; Figure 6C) of BALBc mice compared to non-treated controls (*t*_22_ = 3.647, *p* = 0.0014, Cohen’s *d* = 1.5; Figure 6D). Effects on memory performance, however, were not observed. Both groups of mice required similar time to find the platform (*Treatment: F*_1_, _22_ = 0.7958, *p* = 0.3820; Figure 6A). Unlike in Experiment 3 (cf. Figure 4), this time BALBc mice were impaired in spatial memory and didn’t develop a preference for the target quadrant (Figure 6B; both groups: *F*_3,__487_ = 1.441, *p* = 0.2429; VHC: *F*_2,__023_ = 1.382, *p* = 0.2720; LAC: *F*_1,__872_ = 1.513, *p* = 0.2436; rmANOVA). Moreover, acetyl-L-carnitine treatment did not affect mIns levels in the dorsal hippocampus (dHPC) in subsequent ^1^H-MRS analysis (*t*_19_ = 1.452, *p* = 0.1672, Cohen’s *d* = 0.6; Figure 6E). Thus, treating BALBc mice with acetyl-L-carnitine could not reverse the cognitive deficits.

## Discussion

The present study demonstrates impairments in spatial navigation in BALBc compared to B6N mice. The performance deficits coincided with higher levels of mIns and glial markers, but decreased levels of promnesic molecules and metabolites in the dorsal hippocampus. The increased escape latencies and the molecular changes were not mirrored by deficits in the accuracy of spatial memory suggesting sufficient cognitive reserves to finally master the tasks.

### BALBc Mice Show Deficient Spatial Learning

Compared to B6N, BALBc mice showed higher escape latencies at every timepoint of training both in the WCM and the MWM. There are several explanations for this phenotype: First, BALBc mice might respond to the stressful training procedure with a higher incidence of floating. This is supported by the behavioral data from Experiment 1 and previous reports, where BALBc and B6J mice showed an opposite dependence of floating on the water temperature (Bachli et al., 2008). In fact, BALBc mice are believed to be a highly stress-susceptible strain in general, which is reflected by performance deficits (Francis et al., 1995, 2003; Zaharia et al., 1996; Yoshida et al., 2001; Van Dam et al., 2006) and higher corticosterone levels (Brinks et al., 2007) following stress exposure. Second, higher escape latencies might result from a lack of motivation and/or deficits in executive functioning.

Other than escape latencies, the accuracy of spatial navigation was largely unaffected. The preserved spatial memory is consistent with results of previous lesion studies, which have shown that memory deficits require pronounced neuronal cell loss. In case of rats, preservation of 20–40% of the dHPC is enough to prevent memory deficits in the MWM (Moser et al., 1995). The situation in mice is comparable, whereby preservation of >40% of the hippocampus still enabled spatial learning in the WCM (Kleinknecht et al., 2012). Thus, it remains unclear if BALBc mice can compensate potential memory deficits until the age of 1 year or the behavioral paradigms were just not sensitive enough to detect deficits.

Interestingly, the ability of BALBc mice to develop a spatial memory of the platform position in the MWM varied from experiment to experiment. The volatility of cognitive impairments might be ascribed to the sensitivity of this strain to pre- and postnatal environmental influences (Zaharia et al., 1996; Francis et al., 2003; Thoeringer et al., 2010), which may vary from batch to batch. Together with the impaired accuracy during the second training phase in the WCM (Experiment 2), our findings suggest the BALBc strain as a suitable model strain for studying MCI-like phenomena.

### BALBc Mice Show Higher Myo-Inositol Levels in the Dorsal Hippocampus

By using *in vivo* MRS we were able to assess strain differences for various metabolites in defined brain structures before and/or after spatial training. Most pronounced were the different mIns levels in the dHPC, which were significantly higher in BALBc mice. Noteworthy, BALBc mice showed elevated mIns levels in the dHPC already before the first swimming. After swimming, the differences in mIns were even more pronounced and detectable in the vHPC as well. Follow-up studies will have to include non-trained home cage controls in order to differentiate between the stressful training procedures vs. aging as the main cause of the increased effect sizes observed after training.

### Myo-Inositol – A Marker of Glial Activity?

Elevated mIns levels have been detected in different neurodegenerative disorders (Oz et al., 2010; Sturrock et al., 2015; Wang et al., 2015). In humans, mIns levels were associated with cognitive impairments in Alzheimer’s disease (AD) (Kantarci et al., 2008; Watanabe et al., 2010; Gao and Barker, 2014; Wang et al., 2015) and MCI (Wang et al., 2009; Chen et al., 2016; Franczak et al., 2007). Even though these observations render mIns levels a promising biomarker candidate for the identification of people with upcoming dementia, experimental validation of this approach has been rather unsuccessful so far (Kantarci et al., 2009; Zhang et al., 2015).

Here, we could demonstrate that the increased mIns levels observed in BALBc coincided with increased expression of the microglia marker IBA-1, the astroglial marker GFAP and S100B. IBA-1 is a calcium-binding protein, which is uniformly distributed in microglia (Ito et al., 1998). Interestingly, enhanced microglia activation is suspected to play a key role in the pathogenesis of AD. According to the neuroimmunomodulation theory, activated microglia triggers a pathological cascade, which promotes tau hyperphosphorylation in AD patients (Maccioni et al., 2018). GFAP builds up the cytoskeletal framework of astrocytes (Mondello and Hayes, 2015) whereas S100B is an abundant astroglial calcium-binding protein (Michetti et al., 2019). Transgenic mice overexpressing S100B are strongly susceptible to neuroinflammation (Craft et al., 2005) and showed analogous brain pathology as mouse models of AD (Shapiro et al., 2010). Reduced S100B synthesis, in turn, leads to reduced β-amyloid plaque formation in the brain (Mori et al., 2006). Interestingly, mIns is discussed as a marker of glial activity (Best et al., 2014). Specifically, mIns levels were generally higher in astroglial cells than in neurons (Glanville et al., 1989; Brand et al., 1993), suggesting mIns as an *in vivo* correlate of astrocytic activity.

### Altered Metabolic Pathways in BALBc Mice

We observed marked differences for several metabolites within the dHPC of BALBc and B6N mice. Some metabolites were of particular interest, since they are suspected to play a key role in the pathogenesis of cognitive impairment: the reduced levels of acetylcholine, acetylcarnitine and glutamine. Acetylcholine is thought to play a critical role in cognitive processes and memory function (Ferreira-Vieira et al., 2016). Cholinergic neurotransmission modulates essential neural functions (Ferreira-Vieira et al., 2016). Moreover, the loss of cholinergic neurons in the nucleus basalis of Meynert is a crucial process in the pathogenesis of AD and leads to memory deficits (Whitehouse et al., 1981, 1982). So far, the most common drugs for the treatment of AD patients are cholinesterase inhibitors, which inhibit the breakdown of acetylcholine (Mufson et al., 2008). Glutamine levels reflect an essential part of the glutamate-glutamine cycle and serve as a marker of altered neuron-glia integrity and communication (Xu et al., 2016).

In this study, BALBc mice show reduced levels of acetylcarnitine. Plasma acylcarnitines are suspected to play a key role in brain metabolism (Ciavardelli et al., 2016). Acetylcarnitine is part of the transport of fatty acids into the mitochondria, which is essential for β -oxidation and energy metabolism (Nasca et al., 2018). Interestingly, impaired energy metabolism is suspected to enhance AD-related pathological processes (Chen and Zhong, 2013; Ciavardelli et al., 2016; Johnson et al., 2020). Mitochondrial dysfunctions precede the rise of amyloid plaques and neurofibrillary tangles in preclinical AD models (Yao et al., 2009; Ciavardelli et al., 2016). These metabolic changes are reflected by reduced acetylcarnitine levels in humans suffering from amnestic MCI or AD (Mapstone et al., 2014). The administration of acetylcarnitine is even considered a possible treatment of MCI, since it showed beneficial effects on cognition in several studies with cognitively-impaired patients (Montgomery et al., 2003) and acetylcarnitine is known as precursor of acetylcholine (White and Scates, 1990). In animal models of chronic stress and depression-like symptoms, acetyl-L-carnitine (LAC) treatment diminished stress susceptibility (Cherix et al., 2020) and reversed depression-like behavior (Nasca et al., 2013). Thus, reduced levels of acetylcarnitine in BALBc mice are consistent with the progressing cognitive impairment and associated with AD pathogenesis. Our attempt to revert the performance deficit in the MWM by supplementing the animals with acetylcarnitine via the drinking water, however, failed despite a significant increase in brain levels. Future studies should carefully consider the time point of intervention (e.g., adolescence vs. adulthood), higher doses and prolonged treatment to further investigate therapeutic potentials of acetylcarnitine in BALBc mice.

Our metabolite measurements also revealed a reduction in glutamine levels, which we confirmed by reanalysis of the MRS data. Following its release from excitatory neurons, glutamate is almost exclusively taken up by astrocytes. Here, glutamate is rapidly converted to glutamine and released into the extracellular space, from where it is taken up by neurons and converted to glutamate (Xu et al., 2016). Deficient astrocytic glutamine synthesis has been described in both AD patients and mouse models of AD (Robinson, 2000; Olabarria et al., 2011; Andersen et al., 2020), leading to imbalances in neurotransmitter homeostasis and synaptic functioning (Andersen et al., 2020). Given that glutamine levels directly relate to astrocytic functioning, the reduction in glutamine seems to be at odds with the increase in astrocytic markers (GFAP, S100B). However, the complexity of the interplay between glutamine and glutamate levels does not support a direct relationship between the two measures, without considering differences in glial activity (Xu et al., 2016). Therefore, the changes in glutamine levels support our conclusion of altered astrocyte functioning within the dHPC of BALBc mice.

### What Is “Wrong” With BALBc? Other Neurobiological Peculiarities of BALBc Mice

Previous studies have discovered a neurobiological peculiarity in BALBc mice, which could contribute to the observed performance deficit: BALBc show a coding SNP in the Tryptophan-Hydroxylase-2 (*Tph2*) gene, which results in reduced serotonin content in the brain (Zhang et al., 2004). Serotonin is suspected to play a key role in human cognition (Geldenhuys and Van der Schyf, 2011), and humans, who are impaired in memory consolidation, often have lower serotonin levels than healthy individuals (Cowen and Sherwood, 2013). In post-mortem brains of AD patients, serotonergic neurotransmission is significantly reduced compared to healthy controls (Chen et al., 2000; Hendricksen et al., 2004). Moreover, Tph-2 conditional knockout (*tph2* CKO) AD mice show increased amyloid plaque generation and enhanced density of GFAP-positive astrocytes (Xu et al., 2019). Hence, reduced serotonin levels in the brain caused by a Tph-2 defect may foster neuropathological processes in the brain and, thus, contribute to the performance deficits shown by BALBc mice.

### BALBc – A Model Organism of Mild Cognitive Impairment?

Mild cognitive impairment is defined as a heterogeneous syndrome with a “cognitive decline greater than expected for an individual’s age and education level” (Gauthier et al., 2006). Patients with MCI are still able to handle their daily routine and to preserve general cognitive abilities (Albert et al., 2011). Clinicians differentiate between patients with memory deficits (amnestic MCI) and patients with impairments only in non-amnestic cognitive functions (non-amnestic MCI) (Petersen, 2016). Both subtypes show a higher conversion rate to dementia, e.g., AD, than healthy individuals (Petersen, 2016; see also Michaud et al., 2017). In the present study, BALBc mice showed deficits in behavioral memory tasks, which were largely restricted to performance deficits during training (learning) without consistent impairments in spatial memory. For future investigations, it will be interesting to confirm with alternative tests if BALBc mice are primarily impaired in non-amnestic cognitive domains (e.g., executive functions, complex attention) or develop consistent memory deficits at higher age. It has been shown in previous studies that deficits in executive functions are considered as an early manifestation of AD and typically precede impairments in spatial memory in AD patients (Binetti et al., 1996; Brandt et al., 2009). BALBc mice of this study were tested at an age between eight and 48 weeks, which corresponds to an human age between 20 and 69 years (Flurkey et al., 2007). Consistent memory deficits may emerge at higher age when MCI-like psychopathology may turn into dementia. In humans, the risk of developing dementia increases particularly strongly from the age of 65 up (Van der Flier and Scheltens, 2005).

The alterations in MRS in BALBc mice were analogous to those reported for cognitively impaired mice and humans (Franczak et al., 2007; Chen et al., 2009, 2016; Wang et al., 2009). In contrast, however, BALBc mice failed to show reduced NAA + NAAG levels (Franczak et al., 2007; Wang et al., 2009; Chen et al., 2016), a marker of neural integrity (Castellano et al., 2012). Previous studies revealed that elevated mIns levels are associated with earlier stages of AD in humans, whereas changes of NAA + NAAG levels appear at later stages of AD (Kantarci et al., 2008; Voevodskaya et al., 2016). Moreover, alterations in mIns levels in cognitively impaired humans can even occur in the absence of reduced NAA + NAAG-levels (Kantarci et al., 2000; Catani et al., 2001; Huang et al., 2001). In animal studies with transgenic overexpression of APP/PS1, mIns levels were elevated prior to changes in NAA + NAAG levels (Chen et al., 2009). Thus, mIns is likely a sensitive marker of early neuropathological processes in prodromal stages of cognitive decline. It is of note that we could detect clear differences in mIns levels between the two tested strains, which coincided with performance deficits in BALBc mice. However, there was no direct correlation between mIns levels and performance deficits at the individual level. This may relate to the fact that both BALBc and B6N are inbred strains, that may reduce the interindividual variability of the spectroscopic profiles within each strain.

### Limitations of the Study

We acknowledge that our study has several limitations. First, we did not establish a causal link between mIns, glial cell activity and cognitive deficits. Future studies may be aimed at directly interfering with mIns synthesis (Jupp et al., 2020) to study the consequences on glial cells and spatial memory. Second, we failed to observe consistent deficits in spatial memory in BALBc mice. Possibly, more sophisticated approaches for testing spatial memory or testing older animals (>12 months) might reveal consistent memory impairments.

Third, the applied behavioral paradigms are stressful and metabolically challenging for the animals, which may have initiated or enhanced the observed cognitive deficits. Therefore, future studies should consider using less stressful and less physiological challenging paradigms. Whatsoever, since we observed strain differences in mIns even before any training, we can rule out that those changes simply reflect differences in stress load due to the training procedure. Moreover, they are unrelated to physical and metabolic conditions, since the two strains show similar home cage activity (Fuochi et al., 2021), body weight (Burman et al., 2018), growth rate, body fat (Reed et al., 2007), blood glucose levels and serum lipid profile (Li et al., 2020).

## Conclusion

Our study establishes BALBc mice as a potential model organism of MCI. Moreover, we suggest mIns levels as a prognostic marker of cognitive deficits that are likely associated with alterations in astrocyte activity in the hippocampus.

## Author’s Note

Content of this manuscript has previously appeared online in the dissertation of TE (Ebert, 2021).

## Data Availability Statement

The raw data supporting the conclusions of this article will be made available by the authors, without undue reservation.

## Ethics Statement

The animal study was reviewed and approved by Government of Upper Bavaria (ROB-55.2-2532.Vet_02-17-224).

## Author Contributions

TE, DH, and SA-C performed behavioral experiments. FD and CT performed metabolomics experiments. RC, SA-C, TS, MC, and BB performed and analyzed MRS experiments. KH and NG performed protein work. TE and DH performed pharmacological experiments. TE, FD, TB, NG, OM, FR, SC, and CW analyzed data. CW and TE designed the experiments and wrote the manuscript. CW supervised the project. All authors discussed the results and commented on the manuscript.

## Conflict of Interest

CW was employed by company Boehringer Ingelheim Pharma GmbH & Co. KG. The remaining authors declare that the research was conducted in the absence of any commercial or financial relationships that could be construed as a potential conflict of interest.

## Publisher’s Note

All claims expressed in this article are solely those of the authors and do not necessarily represent those of their affiliated organizations, or those of the publisher, the editors and the reviewers. Any product that may be evaluated in this article, or claim that may be made by its manufacturer, is not guaranteed or endorsed by the publisher.

## Footnotes

1
s-provencher.com/lcmodel.shtml

## References

[B1] Aisen P. Touchon J. Amariglio R. Andrieu S. Bateman R. Breitner J. (2017). EU/US/CTAD Task Force: Lessons Learned from Recent and Current Alzheimer’s Prevention Trials. *J. Prevent. Alzheimer’s Dis.* 116–124. 10.14283/jpad.2017.13 29186281 PMC5724787

[B2] Albert M. S. DeKosky S. T. Dickson D. Dubois B. Feldman H. H. Fox N. C. (2011). The diagnosis of mild cognitive impairment due to Alzheimer’s disease: recommendations from the National Institute on Aging-Alzheimer’s Association workgroups on diagnostic guidelines for Alzheimer’s disease. *Alzheimer’s Dement.* 7 270–279. 10.1016/j.jalz.2011.03.008 21514249 PMC3312027

[B3] Andersen J. V. Christensen S. K. Westi E. W. Diaz-delCastillo M. Tanila H. Schousboe A. (2020). Deficient astrocyte metabolism impairs glutamine synthesis and neurotransmitter homeostasis in a mouse model of Alzheimer’s disease. *Neurobiol. Dis.* 148:105198. 10.1016/j.nbd.2020.105198 33242587

[B4] Annear M. J. Toye C. McInerney F. Eccleston C. Tranter B. Elliott K. E. (2015). What should we know about dementia in the 21st century? A Delphi consensus study. *BMC Geriat.* 15:5. 10.1186/s12877-015-0008-1 25656075 PMC4326452

[B5] Bachli H. Steiner M. A. Habersetzer U. Wotjak C. T. (2008). Increased water temperature renders single-housed C57BL/6J mice susceptible to antidepressant treatment in the forced swim test. *Behav. Brain Res.* 187 67–71. 10.1016/j.bbr.2007.08.029 17923159

[B6] Best J. G. Stagg C. J. Dennis A. (2014). “Chapter 2.5 - Other Significant Metabolites: Myo-Inositol, GABA, Glutamine, and Lactate,” in *Magnetic Resonance Spectroscopy*, eds Stagg C. Rothman D. (San Diego: Academic Press), 122–138.

[B7] Binetti G. Magni E. Padovani A. Cappa S. F. Bianchetti A. Trabucchi M. (1996). Executive dysfunction in early Alzheimer’s disease. *J. Neurol. Neurosurg. Psychiatry* 60 91–93. 10.1136/jnnp.60.1.91 8558161 PMC486198

[B8] Brand A. Richter-Landsberg C. Leibfritz D. (1993). Multinuclear NMR studies on the energy metabolism of glial and neuronal cells. *Dev. Neurosci.* 15 289–298. 10.1159/000111347 7805581

[B9] Brandt J. Aretouli E. Neijstrom E. Samek J. Manning K. Albert M. S. (2009). Selectivity of executive function deficits in mild cognitive impairment. *Neuropsychology* 23 607–618. 10.1037/a0015851 19702414 PMC2769993

[B10] Brinks V. van der Mark M. de Kloet R. Oitzl M. (2007). Emotion and cognition in high and low stress sensitive mouse strains: a combined neuroendocrine and behavioral study in BALB/c and C57BL/6J mice. *Front. Behav. Neurosci.* 1 8–8. 10.3389/neuro.08.008.2007 18958190 PMC2525853

[B11] Burman O. Marsella G. Di Clemente A. Cervo L. (2018). The effect of exposure to low frequency electromagnetic fields (EMF) as an integral part of the housing system on anxiety-related behaviour, cognition and welfare in two strains of laboratory mouse. *PLoS One* 13:54. 10.1371/journal.pone.0197054 29771983 PMC5957419

[B12] Campisi J. Kapahi P. Lithgow G. J. Melov S. Newman J. C. Verdin E. (2019). From discoveries in ageing research to therapeutics for healthy ageing. *Nature* 571 183–192. 10.1038/s41586-019-1365-2 31292558 PMC7205183

[B13] Castellano G. Dias C. S. Foerster B. Li L. M. Covolan R. J. (2012). NAA and NAAG variation in neuronal activation during visual stimulation. *Braz. J. Med. Biol. Res.* 45 1031–1036. 10.1590/s0100-879x2012007500128 22892831 PMC3854159

[B14] Catani M. Cherubini A. Howard R. Tarducci R. Pelliccioli G. P. Piccirilli M. (2001). (1)H-MR spectroscopy differentiates mild cognitive impairment from normal brain aging. *Neuroreport* 12 2315–2317. 10.1097/00001756-200108080-00007 11496102

[B15] Chen C. P. Eastwood S. L. Hope T. McDonald B. Francis P. T. Esiri M. M. (2000). Immunocytochemical study of the dorsal and median raphe nuclei in patients with Alzheimer’s disease prospectively assessed for behavioural changes. *Neuropathol. Appl. Neurob.* 26 347–355. 10.1046/j.1365-2990.2000.00254.x 10931368

[B16] Chen S. Q. Cai Q. Shen Y. Y. Xu C. X. Zhou H. Zhao Z. (2016). Hydrogen Proton Magnetic Resonance Spectroscopy in Multidomain Amnestic Mild Cognitive Impairment and Vascular Cognitive Impairment Without Dementia. *Am. J. Alzheimer’s Dis. Dement.* 31 422–429. 10.1177/1533317515628052 26980718 PMC10852783

[B17] Chen S. Q. Wang P. J. Ten G. J. Zhan W. Li M. H. Zang F. C. (2009). Role of myo-inositol by magnetic resonance spectroscopy in early diagnosis of Alzheimer’s disease in APP/PS1 transgenic mice. *Dement. Geriat. Cogn. Dis.* 28 558–566. 10.1159/000261646 20093832 PMC2837893

[B18] Chen Z. Zhong C. (2013). Decoding Alzheimer’s disease from perturbed cerebral glucose metabolism: Implications for diagnostic and therapeutic strategies. *Prog. Neurob.* 108 21–43. 10.1016/j.pneurobio.2013.06.004 23850509

[B19] Cherix A. Larrieu T. Grosse J. Rodrigues J. McEwen B. Nasca C. (2020). Metabolic signature in nucleus accumbens for anti-depressant-like effects of acetyl-L-carnitine. *Elife* 9:e50631. 10.7554/eLife.50631 31922486 PMC6970538

[B20] Chowdhury S. M. Du X. Tolić N. Wu S. Moore R. J. Mayer M. U. (2009). Identification of cross-linked peptides after click-based enrichment using sequential collision-induced dissociation and electron transfer dissociation tandem mass spectrometry. *Anal. Chem.* 81 5524–5532. 10.1021/ac900853k 19496583 PMC2907912

[B21] Ciavardelli D. Piras F. Consalvo A. Rossi C. Zucchelli M. Di Ilio C. (2016). Medium-chain plasma acylcarnitines, ketone levels, cognition, and gray matter volumes in healthy elderly, mildly cognitively impaired, or Alzheimer’s disease subjects. *Neurobiol. Aging* 43 1–12. 10.1016/j.neurobiolaging.2016.03.005 27255810

[B22] Cowen P. Sherwood A. C. (2013). The role of serotonin in cognitive function: evidence from recent studies and implications for understanding depression. *J. Psychopharm.* 27 575–583. 10.1177/0269881113482531 23535352

[B23] Craft J. M. Watterson D. M. Marks A. Van Eldik L. J. (2005). Enhanced susceptibility of S-100B transgenic mice to neuroinflammation and neuronal dysfunction induced by intracerebroventricular infusion of human beta-amyloid. *Glia* 51 209–216. 10.1002/glia.20194 15810011

[B24] Cummings J. L. Tong G. Ballard C. (2019). Treatment Combinations for Alzheimer’s Disease: Current and Future Pharmacotherapy Options. *J. Alzheimer’s Dis.* 67 779–794. 10.3233/JAD-180766 30689575 PMC6398562

[B25] Ebert T. G. (2021). *On the Search for Translational Biomarkers of Mild Cognitive Impairment*. Ph.D. dissertation. Munich: Technical University Munich.

[B26] Ferreira-Vieira T. H. Guimaraes I. M. Silva F. R. Ribeiro F. M. (2016). Alzheimer’s disease: Targeting the Cholinergic System. *Curr. Neuropharm.* 14 101–115. 10.2174/1570159x13666150716165726 26813123 PMC4787279

[B27] Flurkey K. Currer J. M. Harrison D. E. (2007). “The Mouse in Aging Research,” in *The Mouse in Biomedical Research 2nd Edition. American College Laboratory Animal Medicine*, ed. Fox J. G. (Burlington, MA: Elsevier), 637–672.

[B28] Francis D. D. Szegda K. Campbell G. Martin W. D. Insel T. R. (2003). Epigenetic sources of behavioral differences in mice. *Nat. Neurosci.* 6 445–446. 10.1038/nn1038 12665797

[B29] Francis D. D. Zaharia M. D. Shanks N. Anisman H. (1995). Stress-induced disturbances in Morris water-maze performance: interstrain variability. *Physiol. Behav.* 58 57–65. 10.1016/0031-9384(95)00009-8 7667428

[B30] Franczak M. Prost R. W. Antuono P. G. Mark L. P. Jones J. L. Ulmer J. L. (2007). Proton magnetic resonance spectroscopy of the hippocampus in patients with mild cognitive impairment: a pilot study. *J. Comp. Assist. Tomogr.* 31 666–670. 10.1097/RCT.0b013e318031bc31 17895774

[B31] Fuochi S. Rigamonti M. Iannello F. Raspa M. Scavizzi F. de Girolamo P. (2021). Phenotyping Spontaneous Locomotor Activity in Inbred and Outbred Mouse Strains by Using Digital Ventilated Cages. *Lab. Animal.* 50 215–223. 10.1038/S41684-021-00793-0 34155410

[B32] Gao F. Barker P. B. (2014). Various MRS application tools for Alzheimer disease and mild cognitive impairment. *AJNR Am. J. Neuroradiol.* 35 S4–S11. 10.3174/ajnr.A3944 24742809 PMC4401041

[B33] Garthe A. Kempermann G. (2013). An old test for new neurons: refining the Morris water maze to study the functional relevance of adult hippocampal neurogenesis. *Front. Neurosci.* 7:63. 10.3389/fnins.2013.00063 23653589 PMC3642504

[B34] Gauthier S. Reisberg B. Zaudig M. Petersen R. C. Ritchie K. Broich K. (2006). Mild cognitive impairment. *Lancet* 367 1262–1270. 10.1016/S0140-6736(06)68542-5 16631882

[B35] Geldenhuys W. J. Van der Schyf C. J. (2011). Role of serotonin in Alzheimer’s disease: a new therapeutic target? *CNS Drugs* 25 765–781. 10.2165/11590190-000000000-00000 21870888

[B36] Glanville N. T. Byers D. M. Cook H. W. Spence M. W. Palmer F. B. (1989). Differences in the metabolism of inositol and phosphoinositides by cultured cells of neuronal and glial origin. *Biochim. Biophys.* 1004 169–179. 10.1016/0005-2760(89)90265-8 2546591

[B37] Grady C. (2012). The cognitive neuroscience of ageing. *Nat. Rev. Neurosci.* 13 491–505. 10.1038/nrn3256 22714020 PMC3800175

[B38] Hampel H. Lista S. Teipel S. J. Garaci F. Nisticò R. Blennow K. (2014). Perspective on future role of biological markers in clinical therapy trials of Alzheimer’s disease: a long-range point of view beyond 2020. *Biochem. Pharm.* 88 426–449. 10.1016/j.bcp.2013.11.009 24275164

[B39] Hendricksen M. Thomas A. J. Ferrier I. N. Ince P. O’Brien J. T. (2004). Neuropathological study of the dorsal raphe nuclei in late-life depression and Alzheimer’s disease with and without depression. *Am. J. Psychiatry* 161 1096–1102. 10.1176/appi.ajp.161.6.1096 15169699

[B40] Hering H. Sheng M. (2001). Dendritic spines: structure, dynamics and regulation. *Nat. Rev. Neurosci.* 2 880–888. 10.1038/35104061 11733795

[B41] Huang W. Alexander G. E. Chang L. Shetty H. U. Krasuski J. S. Rapoport S. I. (2001). Brain metabolite concentration and dementia severity in Alzheimer’s disease: a (1)H MRS study. *Neurology* 57 626–632. 10.1212/wnl.57.4.626 11524470

[B42] Ito D. Imai Y. Ohsawa K. Nakajima K. Fukuuchi Y. Kohsaka S. (1998). Microglia-specific localisation of a novel calcium binding protein, Iba1. *Brain Res. Mole. Brain Res.* 57 1–9. 10.1016/s0169-328x(98)00040-0 9630473

[B43] Johnson E. C. B. Dammer E. B. Duong D. M. Ping L. Zhou M. Yin L. (2020). Large-Scale Proteomic Analysis of Alzheimer’s Disease Brain and Cerebrospinal Fluid Reveals Early Changes in Energy Metabolism Associated with Microglia and Astrocyte Activation. *Nat. Med.* 26 769–780. 10.1038/s41591-020-0815-6 32284590 PMC7405761

[B44] Jupp B. Sawiak S. J. van der Veen B. Lemstra S. Toschi C. Barlow R. L. (2020). Diminished Myoinositol in Ventromedial Prefrontal Cortex Modulates the Endophenotype of Impulsivity. *Cerebral Cortex* 30 3392–3402. 10.1093/cercor/bhz317 31897490 PMC7197196

[B45] Kantarci K. Jack C. R. Jr. Xu Y. C. Campeau N. G. O’Brien P. C. Smith G. E. (2000). Regional metabolic patterns in mild cognitive impairment and Alzheimer’s disease: A 1H MRS study. *Neurology* 55 210–217. 10.1212/wnl.55.2.210 10908893 PMC2771162

[B46] Kantarci K. Knopman D. S. Dickson D. W. Parisi J. E. Whitwell J. L. Weigand S. D. (2008). Alzheimer disease: postmortem neuropathologic correlates of antemortem 1H MR spectroscopy metabolite measurements. *Radiology* 248 210–220. 10.1148/radiol.2481071590 18566174 PMC2735577

[B47] Kantarci K. Weigand S. D. Przybelski S. A. Shiung M. M. Whitwell J. L. Negash S. (2009). Risk of dementia in MCI: combined effect of cerebrovascular disease, volumetric MRI, and 1H MRS. *Neurology* 72 1519–1525. 10.1212/WNL.0b013e3181a2e864 19398707 PMC2843530

[B48] Keeler J. F. Robbins T. W. (2011). Translating cognition from animals to humans. *Biochem. Pharm.* 81 1356–1366. 10.1016/j.bcp.2010.12.028 21219876

[B49] Kleinknecht K. R. Bedenk B. T. Kaltwasser S. F. Grunecker B. Yen Y. C. Czisch M. (2012). Hippocampus-dependent place learning enables spatial flexibility in C57BL6/N mice. *Front. Behav. Neurosci.* 6:87. 10.3389/fnbeh.2012.00087 23293591 PMC3530747

[B50] Lanz B. Abaei A. Braissant O. Choi I. Y. Cudalbu C. Henry P. G. (2020). Magnetic resonance spectroscopy in the rodent brain: Experts’ consensus recommendations. *NMR Biomed.* 2020:e4325. 10.1002/nbm.4325 33565219 PMC9429976

[B51] Li J. Wu H. Liu Y. Yang L. (2020). High fat diet induced obesity model using four strainsof mice: Kunming, C57BL/6, BALB/c and ICR. *Exp. Anim.* 69 326–335. 10.1538/expanim.19-0148 32188837 PMC7445062

[B52] Lin A. Andronesi O. Bogner W. Choi I. Y. Coello E. Cudalbu C. (2021). Minimum Reporting Standards for in vivo Magnetic Resonance Spectroscopy (MRSinMRS): Experts’ consensus recommendations. *NMR Biomed.* 34:e4484. 10.1002/nbm.4484 33559967 PMC8647919

[B53] Maccioni R. B. Gonzalez A. Andrade V. Cortes N. Tapia J. P. Guzman-Martinez L. (2018). Alzheimer s Disease in the Perspective of Neuroimmunology. *Open Neurol. J.* 12 50–56. 10.2174/1874205X01812010050 30069256 PMC6040210

[B54] Mapstone M. Cheema A. K. Fiandaca M. S. Zhong X. Mhyre T. R. MacArthur L. H. (2014). Plasma phospholipids identify antecedent memory impairment in older adults. *Nat. Med.* 20 415–418. 10.1038/nm.3466 24608097 PMC5360460

[B55] Michaud T. L. Su D. Siahpush M. Murman D. L. (2017). The Risk of Incident Mild Cognitive Impairment and Progression to Dementia Considering Mild Cognitive Impairment Subtypes. *Dement. Geriat. Cogn. Dis. Extra* 7 15–29. 10.1159/000452486 28413413 PMC5346939

[B56] Michetti F. D’Ambrosi N. Toesca A. Puglisi M. A. Serrano A. Marchese E. (2019). The S100B story: from biomarker to active factor in neural injury. *J. Neurochem.* 148 168–187. 10.1111/jnc.14574 30144068

[B57] Mondello S. Hayes R. L. (2015). “Chapter 16 - Biomarkers,” in *Handbook of Clinical Neurology*, eds Grafman J. Salazar A. M. (Amsterdam: Elsevier), 245–265. 10.1016/B978-0-444-52892-6.00016-7 25702221

[B58] Montgomery S. A. Thal L. J. Amrein R. (2003). Meta-analysis of double blind randomized controlled clinical trials of acetyl-L-carnitine versus placebo in the treatment of mild cognitive impairment and mild Alzheimer’s disease. *Internat. Clin. Psychopharm.* 18 61–71. 10.1097/00004850-200303000-00001 12598816

[B59] Mori T. Town T. Tan J. Yada N. Horikoshi Y. Yamamoto J. (2006). Arundic Acid ameliorates cerebral amyloidosis and gliosis in Alzheimer transgenic mice. *J. Pharm. Exp. Ther.* 318 571–578. 10.1124/jpet.106.105171 16709678

[B60] Morris R. (1984). Developments of a water-maze procedure for studying spatial learning in the rat. *J.Neurosci. Methods* 11 47–60. 10.1016/0165-0270(84)90007-4 6471907

[B61] Moser M. B. Moser E. I. Forrest E. Andersen P. Morris R. G. (1995). Spatial learning with a minislab in the dorsal hippocampus. *Proc. Natl. Acad. Sci. U S A* 92 9697–9701. 10.1073/pnas.92.21.9697 7568200 PMC40869

[B62] Mufson E. J. Counts S. E. Perez S. E. Ginsberg S. D. (2008). Cholinergic system during the progression of Alzheimer’s disease: therapeutic implications. *Exp. Rev. Neurother.* 8 1703–1718. 10.1586/14737175.8.11.1703 18986241 PMC2631573

[B63] Nasca C. Bigio B. Lee F. S. Young S. P. Kautz M. M. Albright A. (2018). Acetyl-l-carnitine deficiency in patients with major depressive disorder. *Proc. Natl. Acad. Sci. U S A* 115 8627–8632. 10.1073/pnas.1801609115 30061399 PMC6112703

[B64] Nasca C. Xenos D. Barone Y. Caruso A. Scaccianoce S. Matrisciano F. (2013). L-acetylcarnitine causes rapid antidepressant effects through the epigenetic induction of mGlu2 receptors. *Proc. Natl. Acad. Sci. U S A* 110 4804–4809. 10.1073/pnas.1216100110 23382250 PMC3607061

[B65] Olabarria M. Noristani H. N. Verkhratsky A. Rodríguez J. J. (2011). Age-dependent decrease in glutamine synthetase expression in the hippocampal astroglia of the triple transgenic Alzheimer’s disease mouse model: mechanism for deficient glutamatergic transmission? *Mole. Neurodegen.* 6:55. 10.1186/1750-1326-6-55 21801442 PMC3199854

[B66] Oz G. Nelson C. D. Koski D. M. Henry P. G. Marjanska M. Deelchand D. K. (2010). Noninvasive detection of presymptomatic and progressive neurodegeneration in a mouse model of spinocerebellar ataxia type 1. *J. Neurosci.* 30 3831–3838. 10.1523/JNEUROSCI.5612-09.2010 20220018 PMC2846771

[B67] Patterson C. (2018). *World Alzheimer Report 2018, The State of the Art of Dementia Research: New frontiers*. London: Alzheimer’s Disease International (ADI).

[B68] Petersen R. C. (2004). Mild cognitive impairment as a diagnostic entity. *J. Inter. Med.* 256 183–194. 10.1111/j.1365-2796.2004.01388.x 15324362

[B69] Petersen R. C. (2016). Mild Cognitive Impairment. *Continuum* 22 404–418. 10.1212/CON.0000000000000313 27042901 PMC5390929

[B70] Pluskal T. Castillo S. Villar-Briones A. Oresic M. (2010). MZmine 2: modular framework for processing, visualizing, and analyzing mass spectrometry-based molecular profile data. *BMC Bioinform.* 11:395. 10.1186/1471-2105-11-395 20650010 PMC2918584

[B71] Reed D. Bachmanov A. A. Tordoff M. G. (2007). Forty mouse strain survey of body composition. *Physiol. Behav.* 91 593–600. 10.1016/j.physbeh.2007.03.026 17493645 PMC2085171

[B72] Reichel J. M. Bedenk B. T. Czisch M. Wotjak C. T. (2017). Age-related cognitive decline coincides with accelerated volume loss of the dorsal but not ventral hippocampus in mice. *Hippocampus* 27 28–35. 10.1002/hipo.22668 27699923

[B73] Reichel J. M. Nissel S. Rogel-Salazar G. Mederer A. Käfer K. Bedenk B. T. (2014). Distinct behavioral consequences of short-term and prolonged GABAergic depletion in prefrontal cortex and dorsal hippocampus. *Front. Behav. Neurosci.* 8:452. 10.3389/fnbeh.2014.00452 25628548 PMC4292780

[B74] Robinson S. R. (2000). Neuronal expression of glutamine synthetase in Alzheimer’s disease indicates a profound impairment of metabolic interactions with astrocytes. *Neurochem. Internat.* 36 471–482. 10.1016/s0197-0186(99)00150-3 10733015

[B75] Ruediger S. Spirig D. Donato F. Caroni P. (2012). Goal-oriented searching mediated by ventral hippocampus early in trial-and-error learning. *Nat. Neurosci.* 15 1563–1571. 10.1038/nn.3224 23001061

[B76] Schroeter M. L. Steiner J. (2009). Elevated serum levels of the glial marker protein S100B are not specific for schizophrenia or mood disorders. *Mole. Psychiatry* 14 235–237. 10.1038/mp.2008.85 19229202

[B77] Shapiro L. A. Bialowas-McGoey L. A. Whitaker-Azmitia P. M. (2010). Effects of S100B on Serotonergic Plasticity and Neuroinflammation in the Hippocampus in Down Syndrome and Alzheimer’s Disease: Studies in an S100B Overexpressing Mouse Model. *Cardiovasc. Psychiatry Neurol.* 2010:153657. 10.1155/2010/153657 20827311 PMC2933893

[B78] Sigurdsson T. Duvarci S. (2015). Hippocampal-Prefrontal Interactions in Cognition, Behavior and Psychiatric Disease. *Front. Syst. Neurosci.* 9:190. 10.3389/fnsys.2015.00190 26858612 PMC4727104

[B79] Sturrock A. Laule C. Wyper K. Milner R. A. Decolongon J. Dar Santos R. (2015). A longitudinal study of magnetic resonance spectroscopy Huntington’s disease biomarkers. *Move. Dis. Offi. J. Move. Dis. Soc.* 30 393–401. 10.1002/mds.26118 25690257

[B80] Thoeringer C. K. Pfeiffer U. J. Rammes G. Pamplona F. A. Moosmang S. Wotjak C. T. (2010). Early life environment determines the development of adult phobic-like fear responses in BALB/cAnN mice. *Genes Brain Behav.* 9 947–957. 10.1111/j.1601-183X.2010.00634.x 20659172

[B81] Tucker A. M. Stern Y. (2011). Cognitive reserve in aging. *Curr. Alzheimer Res.* 8 354–360. 10.2174/156720511795745320 21222591 PMC3135666

[B82] Van Dam D. Lenders G. De Deyn P. P. (2006). Effect of Morris water maze diameter on visual-spatial learning in different mouse strains. *Neurobiol. Learn. Memory* 85 164–172. 10.1016/j.nlm.2005.09.006 16290194

[B83] Van der Flier W. M. Scheltens P. (2005). Epidemiology and risk factors of dementia. *J. Neurol. Neurosurg. Psychiatry* 76:5. 10.1136/JNNP.2005.082867 16291918 PMC1765715

[B84] Voevodskaya O. Sundgren P. C. Strandberg O. Zetterberg H. Minthon L. Blennow K. (2016). Myo-inositol changes precede amyloid pathology and relate to APOE genotype in Alzheimer disease. *Neurology* 86 1754–1761. 10.1212/WNL.0000000000002672 27164711 PMC4862247

[B85] Wang H. Tan L. Wang H. F. Liu Y. Yin R. H. Wang W. Y. (2015). Magnetic Resonance Spectroscopy in Alzheimer’s Disease: Systematic Review and Meta-Analysis. *J. Alzheimer’s Dis* 46 1049–1070. 10.3233/JAD-143225 26402632

[B86] Wang Z. Zhao C. Yu L. Zhou W. Li K. (2009). Regional metabolic changes in the hippocampus and posterior cingulate area detected with 3-Tesla magnetic resonance spectroscopy in patients with mild cognitive impairment and Alzheimer disease. *Acta Radiol.* 50 312–319. 10.1080/02841850802709219 19235582

[B87] Watanabe T. Shiino A. Akiguchi I. (2010). Absolute quantification in proton magnetic resonance spectroscopy is useful to differentiate amnesic mild cognitive impairment from Alzheimer’s disease and healthy aging. *Dement. Geriat. Cogn. Dis.* 30 71–77. 10.1159/000318750 20689286

[B88] Weller J. Budson A. (2018). Current understanding of Alzheimer’s disease diagnosis and treatment. *F1000Res* 7:F1000. 10.12688/f1000research.14506.1 30135715 PMC6073093

[B89] Whalley L. J. Deary I. J. Appleton C. L. Starr J. M. (2004). Cognitive reserve and the neurobiology of cognitive aging. *Ageing Res. Rev.* 3 369–382. 10.1016/j.arr.2004.05.001 15541707

[B90] White H. L. Scates P. W. (1990). Acetyl-L-carnitine as a precursor of acetylcholine. *Neurochem. Res.* 15 597–601. 10.1007/BF00973749 2215852

[B91] Whitehouse P. J. Price D. L. Clark A. W. Coyle J. T. DeLong M. R. (1981). Alzheimer disease: evidence for selective loss of cholinergic neurons in the nucleus basalis. *Ann. Neurol.* 10 122–126. 10.1002/ana.410100203 7283399

[B92] Whitehouse P. J. Price D. L. Struble R. G. Clark A. W. Coyle J. T. Delon M. R. (1982). Alzheimer’s disease and senile dementia: loss of neurons in the basal forebrain. *Science* 215 1237–1239. 10.1126/science.7058341 7058341

[B93] Xu C. J. Wang J. L. Jing P. Min L. (2019). Tph2 Genetic Ablation Contributes to Senile Plaque Load and Astrogliosis in APP/PS1 Mice. *Curr. Alzheimer Res.* 16 219–232. 10.2174/1567205016666190301110110 30827242

[B94] Xu H. Zhang H. Zhang J. Huang Q. Shen Z. Wu R. (2016). Evaluation of neuron-glia integrity by in vivo proton magnetic resonance spectroscopy: Implications for psychiatric disorders. *Neurosci. Biobehav. Rev.* 71 563–577. 10.1016/j.neubiorev.2016.09.027 27702600

[B95] Yao J. Irwin R. W. Zhao L. Nilsen J. Hamilton R. T. Brinton R. D. (2009). Mitochondrial bioenergetic deficit precedes Alzheimer’s pathology in female mouse model of Alzheimer’s disease. *Proc. Natl. Acad. Sci. U S A* 106 14670–14675. 10.1073/pnas.0903563106 19667196 PMC2732886

[B96] Yoshida M. Goto K. Watanabe S. (2001). Task-dependent strain difference of spatial learning in C57BL/6N and BALB/c mice. *Physiol. Behav.* 73 37–42. 10.1016/s0031-9384(01)00419-x 11399292

[B97] Zaharia M. D. Kulczycki J. Shanks N. Meaney M. J. Anisman H. (1996). The effects of early postnatal stimulation on Morris water-maze acquisition in adult mice: genetic and maternal factors. *Psychopharmacology* 128 227–239. 10.1007/s002130050130 8972542

[B98] Zhang B. Ferman T. J. Boeve B. F. Smith G. E. Maroney-Smith M. Spychalla A. J. (2015). MRS in mild cognitive impairment: early differentiation of dementia with Lewy bodies and Alzheimer’s disease. *J. Neuroimag.* 25 269–274. 10.1111/jon.12138 25039916 PMC4295004

[B99] Zhang X. Beaulieu J. M. Sotnikova T. D. Gainetdinov R. R. Caron M. G. (2004). Tryptophan hydroxylase-2 controls brain serotonin synthesis. *Science* 305:217. 10.1126/science.1097540 15247473

[B100] Zhang Z. Ma Z. Zou W. Guo H. Liu M. Ma Y. (2019). The Appropriate Marker for Astrocytes: Comparing the Distribution and Expression of Three Astrocytic Markers in Different Mouse Cerebral Regions. *BioMed. Res.* I*nternat.* 2019:9605265. 10.1155/2019/9605265 31341912 PMC6613026

