# Supplementary material for: Myo-Inositol Levels in the Dorsal Hippocampus Serve as Glial Prognostic Marker of Mild Cognitive Impairment in Mice
Source: Front Aging Neurosci. 2021 Nov 12;13:731603. doi: 10.3389/fnagi.2021.731603 (PMC8633395; doi:10.3389/fnagi.2021.731603)
Supplement: Supplementary file 1 [file Data_Sheet_1.PDF]

**1.**	**Hardware**	
a.	Field strength [T]	9.4T
b.	Manufacturer	BRUKER
c.	Model (software version if available)	Biospec 94/20 system, ParaVision 6.0.1
d.	RF coils: nuclei (transmit/receive), number of channels, type, body part	i) two channel transmit/receive cryo coil<br>ii) whole body coil (transmission) / room temperature 2x2 array surface coil (detection)
e.	Additional hardware	-
**2.**	**Acquisition**	
a.	Pulse sequence	PRESS
b.	Volume of interest (VOI) locations	dorsal and ventral hippocampus
c.	Nominal VOI size [cm3, mm3]	2.5 × 1.5 × 1.5 mm^3
d.	Repetition time (TR), echo time (TE) [ms, s]	TR=5000 ms; TE=16.5 ms
e.	Total number of excitations or acquisitions per spectrum<br>In time series for kinetic studies<br>i. Number of averaged spectra (NA) per time point<br>ii. Averaging method (eg block-wise or moving average)<br>iii. Total number of spectra (acquired/in time series)	128 averages
f.	Additional sequence parameters (spectral width in Hz, number of spectral points, frequency offsets)<br>If STEAM:, mixing time ™<br>If MRSI: 2D or 3D, FOV in all directions, matrix size, acceleration factors, sampling method	4041.41 Hz, 2048 points
g.	Water suppression method	
h.	Shimming method, reference peak, and thresholds for “acceptance of shim” chosen	MAPSHIM
i.	Triggering or motion correction method (respiratory, peripheral, cardiac triggering, incl. device used and delays)	-
**3.**	**Data analysis methods and outputs**	
a.	Analysis software	LCModel 6.3
b.	Processing steps deviating from quoted reference or product	-
c.	Output measure (eg absolute concentration, institutional units, ratio), processing steps deviating from quoted reference or product	ratio to total creatine
d.	Quantification references and assumptions, fitting model assumptions	LCModel default basis
**4.**	**Data quality**	
a.	Reported variables (SNR, linewidth (with reference peaks))	see suppl. table 1
b.	Data exclusion criteria	CRLB>14%, FWHM>0.118ppm, SNR<7 (cryocoil) / SNR<3 (room temp. coil)
c.	Quality measures of postprocessing model fitting (eg CRLB, goodness of fit, SD of residual)	see 4b
d.	Sample spectrum	see suppl. figure 1
