# Supplementary material for: Myo-Inositol Levels in the Dorsal Hippocampus Serve as Glial Prognostic Marker of Mild Cognitive Impairment in Mice
Source: Front Aging Neurosci. 2021 Nov 12;13:731603. doi: 10.3389/fnagi.2021.731603 (PMC8633395; doi:10.3389/fnagi.2021.731603)
Supplement: Supplementary file 2 [file Data_Sheet_2.docx]

Supplementary Materials


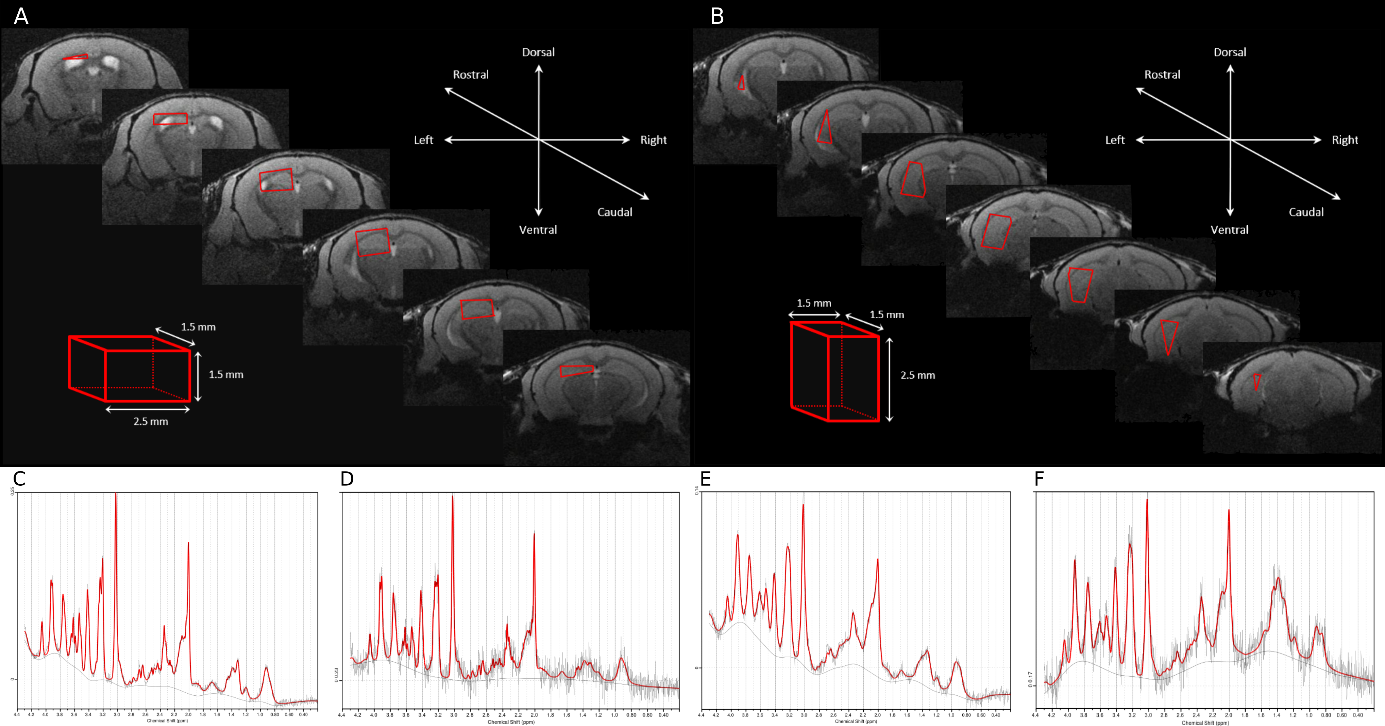


Suppl. Figure 1: Voxel positioning for the MRS experiments, along with representative MR spectra: A) dorsal hippocampus (dHPC), B) ventral hippocampus (vHPC). The voxel position is highlighted in red, and overlayed to the T2-weigthed coronal slices. C) and E) dHPC and vHPC spectra acquired with the cryocoil. D) and F) dHPC and vHPC spectra acquired with the room temperature coil. Blue arrows highlighting the peaks of myo-inositol, green arrows highlighting the peak of N-acetylaspartate and N-acetylaspartateglutamate.


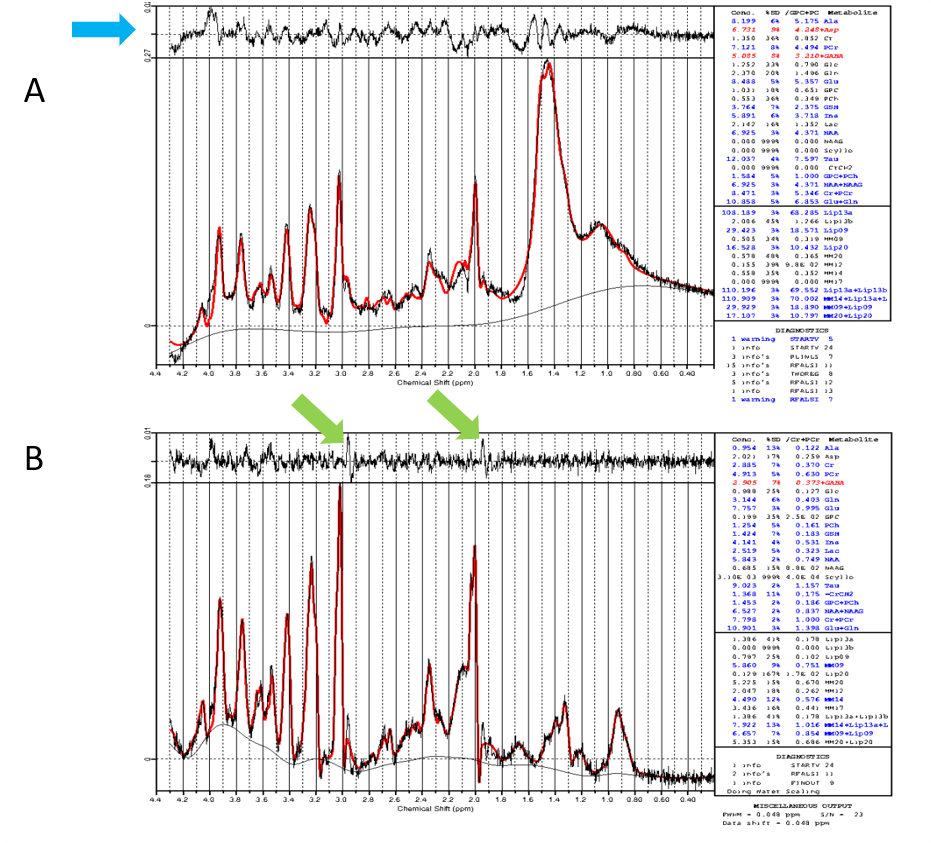


Suppl. Figure 2: Examples of failed metabolite fits, which were excluded from further analysis. Despite excellent signal-to-noise, narrow linewidth and small CRLB% for the individual metabolites, metabolic fitting was not successful. A) Strong residual signals across the whole spectral window (blue arrow). B) Isolated residual peaks (green arrow) close the tCR and NAA+NAAG frequencies, likely falsifying the estimated metabolic concentrations.

Suppl. Table 1: Descriptive statistics for FWHM and SNR per measurement

|  |  |  | FWHM (ppm) | | | SNR | | |  |
| --- | --- | --- | --- | --- | --- | --- | --- | --- | --- |
|  |  |  | mean | min | max | mean | min | max |  |
| Experiment 1 | MRS 1 | dHPC | 0.040 | 0.021 | 0.097 | 22.2 | 9 | 54 | cryocoil |
| Experiment 2 | MRS 2a | dHPC | 0.034 | 0.021 | 0.070 | 21.7 | 13 | 63 | cryocoil |
|  |  | vHPC | 0.055 | 0.027 | 0.097 | 14.7 | 7 | 24 | cryocoil |
| Experiment 2 | MRS 2b | dHPC | 0.033 | 0.019 | 0.054 | 8.2 | 5 | 12 | room temp. coil |
|  |  | vHPC | 0.049 | 0.027 | 0.118 | 4.9 | 3 | 8 | room temp. coil |
| Experiment 4 | MRS 3 | dHPC | 0.035 | 0.021 | 0.094 | 24.9 | 14 | 37 | cryocoil |
